# Design and function of targeted endocannabinoid nanoparticles

**DOI:** 10.1038/s41598-022-21715-1

**Published:** 2022-10-14

**Authors:** N. Barrie, N. Manolios, J. Stuart, T. Chew, J. Arnold, R. Sadsad, L. De Campo, R. B. Knott, J. White, D. Booth, M. Ali, M. J. Moghaddam

**Affiliations:** 1 Faculty of Medicine and Health, The University of Sydney, Sydney, NSW 2006 Australia; 2 Department of Rheumatology, Westmead Hospital, Westmead, Sydney, NSW 2145 Australia; 3 Discipline of Pharmacology, The University of Sydney, School of Psychology, Sydney, Australia; 4 Lambert Initiative for Cannabinoid Therapeutics, Brain and Mind Centre, The University of Sydney, Sydney, NSW 2006 Australia; 5 Sydney Informatics Hub, Core Research Facilities, The University of Sydney, Sydney, 2006 Australia; 6 Small-Angle Neutron Scattering (SANS) Facility, Australian Nuclear Science and Technology Organisation (ANSTO), Lucas Heights, Sydney, NSW 2234 Australia; 7 Materials Ccharacterization and Modelling, Commonwealth Scientific and Industrial Research Organisation (CSIRO), Manufacturing, Clayton, VIC 3169 Australia; 8 Medicine, Westmead Clinical School, The Westmead Institute for Medical Research, Westmead, Sydney, NSW 2145 Australia; 9 Professor of Medicine, The University of Sydney, Sydney, NSW 2006 Australia; 10 The University of Sydney, Sydney, NSW 2006 Australia; 11 CSIRO, Manufacturing Flagship, North Ryde, Sydney, NSW 2113 Australia; 12 Present Address: Co-CEO Nanomed Pty Ltd. Unit2, 11-13 Orion Road, Lane Cove, Sydney, NSW 2066 Australia

**Keywords:** Biotechnology, Rheumatology, Nanoscience and technology

## Abstract

Nanoparticles and nano-delivery systems are constantly being refined and developed for biomedical applications such as imaging, gene therapy, and targeted delivery of drugs. Nanoparticles deliver beneficial effects by both release of their cargo and by liberation of their constitutive structural components. The *N-*acylethanolamines linoleoyl ethanolamide (LEA) and oleoyl ethanolamide (OEA) both exhibit endocannabinoid-like activity. Here, we report on their ability to form nanoparticles that when conjugated with tissue-specific molecules, are capable of localizing to specific areas of the body and reducing inflammation. The facilitation of pharmacological effects by endocannabinoids at targeted sites provides a novel biocompatible drug delivery system and a therapeutic approach to the treatment, patient management and quality of life, in conditions such as arthritis, epilepsy, and cancer.

## Introduction

Endogenous cannabinoids belong to a group of molecules known as *N-*acylethanolamines (NAEs) which are naturally occurring body lipids that are involved in many physiological and metabolic processes^1^. Of the NAEs, anandamide, a biochemically important lipid molecule, is the most widely characterized. It maintains basal endocannabinoid biological activity by binding to cannabinoid receptor 1 (CB_1_) and cannabinoid receptor 2 (CB_2_)^2^, as well as, the transient receptor potential vanilloid 1 (TRPV1) protein^3^, responsible for the integration of noxious stimuli causing pain^4^. While the anti-inflammatory effects of anandamide are well documented^5,6^, the rapid cellular degradation by fatty acid amide hydrolase (FAAH) limits its role and efficacy in vivo^7,8^. Other lesser-known NAEs such as linoleoylethanolamide (LEA), oleoylethanolamide (OEA), stearoylethanolamide (SEA) and palmitoylethanolamide (PEA) have recently gained attention due to their anti-inflammatory and analgesic properties^5,6,9^ and may be viewed as therapeutic alternatives to anandamide. Unlike anandamide, these compounds have limited or no affinity for the CB_1_ and CB_2_ receptors reducing psychotropic side-effects but contributing to anti-inflammatory results by regulation through the “entourage” effects^10,11^. LEA has been shown to inhibit FAAH^12^ thereby extending the life of anandamide^13,14^ and enhancing the therapeutic potential in inflammatory-based diseases. Similarly, OEA exerts its potent anti-inflammatory effects by enhancing peroxisome proliferator activating receptor (PPARα) expression^15^ that reduces the production of pro-inflammatory cytokines. The reduced nociceptive effects of OEA, are mediated through the glutamate-signalling pathway^9^. Overall, the endocannabinoid system has a valuable role to play in inflammation through the regulation of immune cell migration, cytokine production and nociceptive transmission.

Based on our previous studies^16–18^ examining the lyotropic mesophase behaviour of mixed NAE lipids to self-assemble, we noted that various mixtures of LEA and OEA were able to form a variety of nanoparticles (NPs). In the present study, a systematic investigation of LEA and OEA indicated that 40% OEA:60% LEA was the optimum ratio for forming stable liquid crystalline mesophases and stable NPs in an aqueous solution at physiological temperatures. Polyethylene glycol (PEG)-oleoyl, conjugated to a synovium-targeting peptide (HAP-1)^19^ was incorporated within the membrane of the NP, then injected into adjuvant induced arthritic rats to investigate localization and therapeutic outcomes. Here we outline the method of NAE synthesis, and NP generation, characterization, localization, and mechanism of action, and discuss potential future applications.

## Materials and methods

### Materials

Organic solvents were purchased from Merck (Victoria, Australia) and were either analytical or spectroscopic grade and used as received. Ethanolamine and other reagents were purchased from Sigma-Aldrich (Sydney, Australia). Oleic acid and linoleic acid were purchased from Nu Check Prep (Minnesota, USA). Polyethylene glycol-2000 (PEG2000) was obtained from Badische Anilin- & Sodafabrik (BASF, Victoria, Australia). Targeting peptides HAP-1 (sequence: SFHQFARATLAS), and scrambled HAP-1 (sHAP-1; sequence: ALSRAFSHFQTA)^19^ were custom synthesized by Auspep (Victoria, Australia). Fluorescent lipid DiD (1,1,dioctadecyl-3,3,3′,3′-tetramethylindodicarbocyanine, 4-chlorobenzenesulfonate salt) was purchased from Molecular Probe (Invitrogen, Melbourne, Australia). Purchased peptides were shown to be of high purity (> 80%) by Reverse Phase High Performance Liquid Chromatography (RP-HPLC) and characterized by a Mass Spectrometer (MS) equipped with a matrix assisted laser desorption/ionization (MALDI) source (Applied Biosystems Inc., Foster City, CA, USA).

### Synthesis and characterization of NPs

#### Monoethanolamide lipid synthesis

Monoethanolamide lipids, OEA and LEA were synthesized and purified to > 99% purity as previously reported^17^. The PEGylated lipid (Ole-PEG2000-Succ) was synthesized in a two-step procedure and the synovia-targeted peptide HAP-1 and the corresponding scrambled sequence, sHAP-1, were conjugated to the distal end of the PEG group. Details and methods for the synthesis of Ole-PEG2000-Succ and linkage to targeting peptides HAP-1 and sHAP-1 are provided in Supplementary Materials and Methods.

#### Peptide purification

Peptides were initially dissolved in Milli-Q water containing 0.05% TFA. If insoluble, a drop-wise addition of acetonitrile/water (9:1, v/v), containing 0.05% TFA continued until the peptides dissolved. Purification of samples was carried out using a semi-preparative C18-WP 12 g column (Reveleris, Grace, Victoria, Australia) on an Agilent 1100 series system (Agilent, Melbourne, Australia; flow rate 30 mL/min^−1^) and using a linear gradient as described below of solvent A: 80% v/v water containing 20% v/v acetonitrile, and solvent B: 60% v/v THF and 40% v/v acetonitrile. The purity of the fractions was monitored by analytical reverse phase-HPLC using a C18 analytical column (Vydac; 4.5 mm × 250 mm, flow rate 1 mL/min^−1^; gradient 1% B min^−1^). Mass spectrometry was performed over the appropriate mass/charge (*m/z*) range on a QSTAR XL mass spectrometer equipped with a MALDI source (Applied Biosystems Inc., Foster City, CA, USA). Mass spectroscopy scans of synthesized peptides are shown in Supplementary Fig. 1A–C. Further, peptide purity assessment is outlined in Supplementary Materials and Methods.

#### Differential scanning calorimetry (DSC)

DSC was performed using a Mettler Toledo DSC 822 system equipped with a Mettler TSO 801RO sample robot (Mettler Toledo; Melbourne, Australia). 5–10 mg of sample was placed in aluminium crucibles and cooled to − 130 °C before heating at a rate of 2.5 °C min^−1^ up to 120 °C. Thermal calibration of the ceramic sensor was performed by integration of a standard indium sample. DSC thermograms of the monoethanolamide mixes were recorded using the STARe software package.

#### Water penetration scans

Direct observation of the mesophase birefringence via cross-polarizing optical microscopy (POM) provided a simple and rapid assessment of the lyotropic phase behaviour of the mixed amphiphilic system. Samples of monoethanolamide amphiphile mixtures of increasing OEA to LEA ratio/content were combined in an ethanol solution, vortexed, and evaporated to dryness, using a rotary evaporator. Dried samples were then freeze-dried overnight. A small amount of mixed monoethanolamide amphiphile was placed onto a microscope slide and heated to melt on a hot stage to achieve an even lipid surface. A coverslip was placed on top of the melted amphiphile and then cooled to room temperature prior to addition of water. Water placed at the edges of the coverslip was drawn between the two glass surfaces to surround the solidified material by capillary action. The interaction of water and the monoethanolamide amphiphile at 25 °C and 37 °C was observed with an Olympus GX51 inverted optical microscope (Olympus Australia Pty. Ltd., Melbourne, Australia) in the presence and absence of polarizing lenses. The temperature of the sample mounted on a Linkam hot stage was controlled by a Linkam control processor PE94 (Linkam PE 94, Linkam Scientific Instruments Ltd, Surrey, UK). Images were captured with an Olympus C-5060 digital camera (Olympus Australia Pty. Ltd, Melbourne, Australia).

#### Small angle X-ray scattering (SAXS)

Small angle X-ray scattering measurements were used for definitive phase assignment, and to obtain lattice parameters, sampling regions of interest determined from the partial binary phase diagram. Samples were made up to 70% excess water content by adding a known volume of HPLC-grade water to the pre-weighed dry lipid. To ensure homogeneity, samples were allowed to equilibrate for a period of no less than 24 h. SAXS analysis of bulk and lyotropic mesophases at excess water (70 wt%) were performed using a Nano STAR SAXS instrument (Bruker, Karlsruhe, Germany). The SAXS instrument was equipped with a Peltier controlled sample stage. The Bruker software was used to correct the 2D SAXS patterns and produce 1D profiles for further analysis. Scattering intensities, I(q) was plotted as a function of the scattering vector q, where q = (4π/λ) sin(θ/2), in which λ is the wavelength and θ is the scattering angle. Liquid crystalline mesophases gave rise to distinct diffraction patterns that were used as an unambiguous identification of each phase.

#### Dynamic light scattering (DLS)

The physical characterization of the NPs was carried out using a Zetasizer Nano ZS (Malvern Instruments, Worcestershire, UK) equipped with a photon correlation spectrometer. Measurements were performed at 25 °C and the scattered light was detected at a scattering angle of 90°. Particle size was determined by an intensity-weighted mode and averaged over three measurements, with each measurement averaged over 14 scans.

#### Cryogenic transmission electron microscopy (cryo-TEM)

Cryo-TEM was employed to visualize the nanostructure of the dispersed mesophases. Samples were sonicated for 10 min prior and vortexed for 10 s immediately before plunging. Droplets (4 μL) of NP suspensions were placed onto a 300-mesh copper grid coated with lacy formvar-carbon film (Pro- SciTech, Queensland, Australia) and gently blotted with filter paper to obtain a thin liquid film (20–400 nm). Following adhesion of NPs, the grid was plunged into ethane cooled by liquid nitrogen. The sample was then transferred to a Cryo-TEM. Photomicrographs were acquired using Tecnai 12 Biotwin TEM (FEI Co., Eindhoven, Netherlands) at an operating voltage of 120 kV, equipped with an FEI Eagle 4 k × 4 k CCD (FEI, Eindhoven, the Netherlands). Samples were viewed at a range of magnifications.

#### Generation of NP’s

OEA and LEA were combined in a 4:6 ratio, and dissolved in ethanol forming a thin lipid layer after drying the solvent in a rotary evaporator (Rotavapor R-210; Buchi Instruments, Germany) at 40 °C. For the incorporation of the (PEG) stabilizers, the lipid film was then hydrated with a 15% (w/v) Ole-PEG2000-OH in PBS solution and sonicated at 35 °C for 1 h with intermittent probe-sonication homogenization (Benchmark D1000 Homogenizer, PathTech, Australia) to allow hydration of the sample and formation of NP dispersions. For HAP-1 targeted-nanoparticles (NP_HAP-1_) and sHAP-1 targeted-nanoparticles (NP_sHAP-1_), 7% (w/v) Ole-PEG2000-HAP-1 or Ole-PEG2000-sHAP-1 respectively, were dissolved in ethanol and incorporated into the phospholipid membrane via rotary evaporation prior to the hydration and sonication of lipid layers. For fluorescent NPs, lipophilic fluorochrome tracer DiD was incorporated into the lipid membrane of the NP. This was achieved by adding 1% (w/v) DiD to the 40%OEA:60%LEA mix before dissolving the mixture in ethanol and removal of the solvent in the rotary evaporator at RT as previously described. Assessment of the NPs size, morphology and nanostructure using Nanosizer, cryo-TEM and SAXS analyses, are shown respectively in Supplementary Fig. 2A–C.

### In-vitro studies

#### Cell culture

Human-derived fibroblast-like synoviocytes (h-FLS), derived from rheumatoid arthritis synovium [rheumatoid arthritis-FLS (RA-FLS)] or, synovium of osteoarthritis patients [osteoarthritis-FLS (OA-FLS)] were purchased from American Type Culture Collection (ATCC) and cultured and maintained in synoviocyte growth medium (Cell Applications Inc, San Diego, CA, USA) at 37 °C and 5% CO_2_. Rabbit fibroblast like synoviocytes (HIG-82) were cultured in Ham’s F12 medium (Gibco, Thermo Fischer Scientific, Waltham, MA, USA) supplemented with 2 mM l-glutamine, 50 units/mL penicillin, 50 µg/mL streptomycin (Invitrogen, Melbourne, Australia) and 10% heat-inactivated foetal bovine serum (Sigma-Aldrich, Castle Hill, Australia) at 37 °C and 5% CO_2_. The cells used were from early passages (passages 3–7). For confocal studies, FLS and HIG-82 cells were seeded at 3 × 10^4^ cells onto glass coverslips and incubated overnight at 37 °C. For flow cytometry studies, h-FLS cells (passage: 4) were seeded at a density of 5 × 10^5^ into six well plates (Corning, Sigma Aldrich, NSW, Australia) and grown to confluence prior to treatment.

#### WST-assay

WST-1, a tetrazolium dye, was used as a colorimetric assay to assess cell viability. NP cytotoxicity was assessed using WST-1 assay (Quick Cell Proliferation Assay Kit II, Mountain View, CA, USA), and performed according to the manufacturer’s instructions. Further details are described in Supplementary Materials and Methods.

#### Immunofluorescence of HAP-1 binding

HAP-1-biotin and sHAP-1-biotin were incubated with streptavidin-FITC. The biotinylated-streptavidin conjugates were then incubated with FLS cells (normal, RA and OA) at 37 °C for 3 h. Following incubation, the slides were washed with washing buffer (PBS, 1% FCS) to remove non-specific staining and fixed with 2% paraformaldehyde for 20 min on ice. Slides were again washed, and the actin cytoskeleton stained using TRITC-phalloidin (1:2000 dilution). After washing, DAPI was used to stain the cell nuclei. Slides were then washed and embedded in mounting medium FluorSave Reagent (Calbiochem, San Diego, CA, USA). Images of cells were captured using an Olympus FV confocal laser scanning microscope (Olympus, Victoria, Australia).

#### Fluorescence assisted cell sorting (FACS)

Flow cytometry was used to assess the NP in vitro cellular binding in culture. h-FLS (passage 4) and HIG-82 cells were seeded at a density of 5 × 10^5^ into six well plates and grown to confluence. After 48 h, the medium was replenished with medium containing 30 μg/mL of fluorescently labelled NP_non-targeted_ or NP_HAP-1_. Cells were then incubated for 1, 3 and 18 h at 37 °C. For assessment of NP dye leakage, NP were incubated with h-FLS cells for 3 h at 4 °C. Untreated cells and free DiD dissolved in DMSO were used as a control. Following incubation, the medium was removed, and the cells washed three times with FACS buffer (PBS supplemented with 1% BSA) to remove surface-associated NPs. Cells were then detached using trypsin/EDTA (0.5 mM) and fixed in 1 mL of 4% paraformaldehyde in PBS solution for 1 h. The stained and fixed cells were stored in the dark at 4 °C and the NP uptake measured as fluorescence intensity was analyzed on a flow cytometer. The method used for quantification of NP-cell complexes by flow cytometry is outlined in Supplementary Materials and Methods.

#### OEA and LEA effects on cytokine production

RT-PCR was carried out to investigate whether NP treatment could regulate tumor necrosis factor (TNF)-α stimulated changes at the gene transcription level of three target genes: nuclear factor kappa-light-chain-enhancer of activated B cells (NF-κβ), interleukin (IL)-6 and IL-8. Changes in gene regulation are expressed as a fold change to control groups and normalized against glyceraldehyde 3-phosphate dehydrogenase (GAPDH). Consistent with previously documented pro-inflammatory actions of TNF-α, incubation of cultured RA-FLS with recombinant TNF-α (10 ng/mL) induced marked increases in the gene transcription for all three genes tested.

### In vivo studies

#### Animals

For in vivo localization experiments, rats were housed in the Kolling Institute Kearn’s Animal Facility located within Royal North Shore Hospital, Sydney, and imaging performed using their in-house near infrared (NIR) facility. All other in vivo work was performed at the Westmead Housing facility (Westmead Institute for Medical Research, WMIR) under the appropriate ethics approval. Further description is included in Supplementary Materials and Methods.

#### Adjuvant-induced arthritis (AIA)

Arthritis was induced by subcutaneous injection (SCI) of lyophilized *Mycobacterium tuberculosis* (MTB) suspended in 100 μL of squalene into the base of the tail of the individual rats, as previously described by Manolios et al.^20^. The other group was used as a control. On average, arthritis developed 11–14 days post MTB injection. During this time, rats were monitored for pain and changes in wellbeing and buprenorphine was given when signs of pain or distress were shown. Rats were deemed to be arthritic when both redness and swelling were present in the same joint(s). On the second day, rats were anesthetized prior to injection of NPs under isoflurane/oxygen (2% v/v isoflurane in O_2_1L/min).

#### NP body distribution

Fluorescently labelled NP_non-targeted_, NP_HAP-1_ and NP_sHAP-1_ were intravenously administered into the tail vein of each animal, followed by a flush of 300 µL saline injection. The animals remained anesthetized and were placed in a prone position on a gamma camera (double head; Siemens Medical Systems, IL, USA) equipped with a low-energy high-resolution collimator. NP in-vivo localization was captured at 1, 3, 24 and 48 h for 10 min using a Bruker FT-NIR imager. For optimum DiD intensity, emission was taken at 700 nm with a corresponding excitation of 650 nm. ‘Image J’-computer imaging program was used on the region of interest (ROI) on the chosen arthritic joint of each rat for each time-point and compared to the background fluorescence at the same ROI of the same rat. The values reported were obtained by first averaging the signal value determined in the joints (n = 3) then by subtracting its background value from the signal obtained at each time-point for each joint.

#### NP concentrations in tissue

Arthritic rats were anesthetized under isoflurane/oxygen (2% v/v isoflurane in 1 L/min O_2_) and the NPs injected intravenously via the tail vein. The treatment groups were as follows; untreated control (n = 5), vehicle control (n = 5), NP_non-targeted_ (n = 5) and NP_HAP-1_ (n = 5). Following injection, blood samples were collected via a temporary cannula in the lateral tail vein at various time intervals (0, 45 min, and 1.5, 3, and 6 h). Collected blood was spun down and the rat plasma separated, snap frozen in liquid nitrogen (LN_2_) and stored at − 80 °C for analysis. After 6 h the animals were sacrificed and the kidneys, liver, spleen, lungs and paws were harvested, snap frozen in LN_2_ and stored at − 80 °C for analysis. Lipid extractions were adapted from Stuart et al.^21^. Further tissue processing methods are outlined in Supplementary Materials and Methods.

#### Quantification of endocannabinoids in rat serum by HPLC/MS/MS

20 µL injections of each sample were rapidly separated using a C8 Zorbax guard column in conjunction with a C18 Zorbax reverse-phased analytical column by a gradient of 20% ultrapure HPLC MeOH, 80% filtered HPLC water with 1 mM ammonium acetate (mobile phase A) and 100% ultrapure HPLC grade MeOH and 1 mM ammonium acetate (mobile phase B). Two Shimadzu LC-30AD pumps (Rydalmere, NSW, Australia) were then used to create a pressurised gradient elution (200 µL/min). A Shimadzu 8030 triple quadrupole MS was used to ionize the sample using positive electrospray ionization (ESI) through a multiple reaction monitoring method. Synthetic standards of PEA, OEA, LEA and d4-AEA (Cayman Chemical, Ann Arbor, MI, USA) were used to generate calibration curves for quantification by LabSolutions software (Shimadzu, Rydalmere, NSW, Australia). The concentration of each analyte was then converted to moles per gram tissue (using the weights obtained). Statistical analysis was performed using GraphPad Prism (Version 7).

#### Quantification of inflammatory cytokines in circulating rat plasma

Arthritic rats were intravenously injected with NPs and blood plasma concentrations of cytokines—IL-1α, IL-1β, IL-2, IL-4, IL-6, IL-10, granulocyte–macrophage colony stimulating factor (GM-CSF), interferon (IFN)-γ and TNF-α, assessed using a LEGENDplex bead-based immunoassay and quantified by flow cytometry. Arthritic rats were divided into four groups of three each and treated as follows: untreated control (ART-CON), NP_non-targeted_ (ART-NP_non-targeted_) and NP_HAP-1_ (ART-NP_HAP-1_). NPs were intravenously administered once a day, for two days, for a total of two injections. The control group received two injections of normal saline in the same volume. Blood was collected by the lateral tail vein 48 h after the initial injection. Further description is included in Supplementary Materials and Methods.

### RNA sequencing (RNA-SEQ)

Detailed methods for stimulation of human RA-FLS cells, RNA isolation, RNA-seq construction, RT-PCR and gene expression analysis are outlined in the Supplementary Materials and Methods. Stranded RNA libraries were prepared from 350 ng RNA using the Illumina^®^ TruSeq Stranded mRNA Prep kit. The Australian Genome Research Facility sequenced the libraries on an Illumina HiSeq 2500 to generate 50 base-pair (bp) single-end reads. Raw sequencing reads were assessed for quality using FastQC (version 0.11.3) (Babraham Bioinformatics), ensuring that Phred scores were over 30. Per base quantity scores were confirmed to be high using FastQC, and therefore no adaptor trimmings were performed. STAR (version 2.5.2a) was used to align the reads to Release 19 of the human reference genome (GRCh37/hg19), with the GENCODE (Release 28) annotation (http://www.gencodegenes.org/) provided, using default parameters. Resulting SAM files were sorted by position using SAMtools (version 1.6). Quality assessment, mapping and raw read counts were conducted on the high-performance computing cluster (Artemis), provided by the Sydney Informatics Hub, University of Sydney.

Illumina RNA-seq was performed to investigate the effect of NPs on gene expression using human RA-FLS cells. Treatment groups were performed in three biological replicates and included untreated cells (RA-UT), NP treated cells (RA-NP), TNF-α stimulated cells (RA-TNF) and TNF-α stimulated cells with NP treatment (RA-TNF/NP). To investigate NP gene regulation, RA-FLS were incubated with NPs alone (RA-NP) or in the presence of TNF-α (RA-TNF/NP) and molecular relationships assessed using RNA-seq. Quality analysis statistics of RNA-seq data are summarized in Supplementary Table 1. Replicates within each of the four treatment groups could be clearly distinguished for RA cells using principal component analysis (PCA) plots and sample-sample distance heat maps provided by DESeq2 (Supplementary Fig. 5). For RA-FLS cells, the number of clusters, along with the percentage variance between the clusters was reported and confirmed that the samples were clustered within their respective treatment groups, as expected. Statistical analysis of differentially expressed (DE) genes was carried out using the Ingenuity Pathway Analysis (IPA) program applying a 5% false discovery rate (FDR).

## Results

### Characterization of NPs

**Figure 1:**
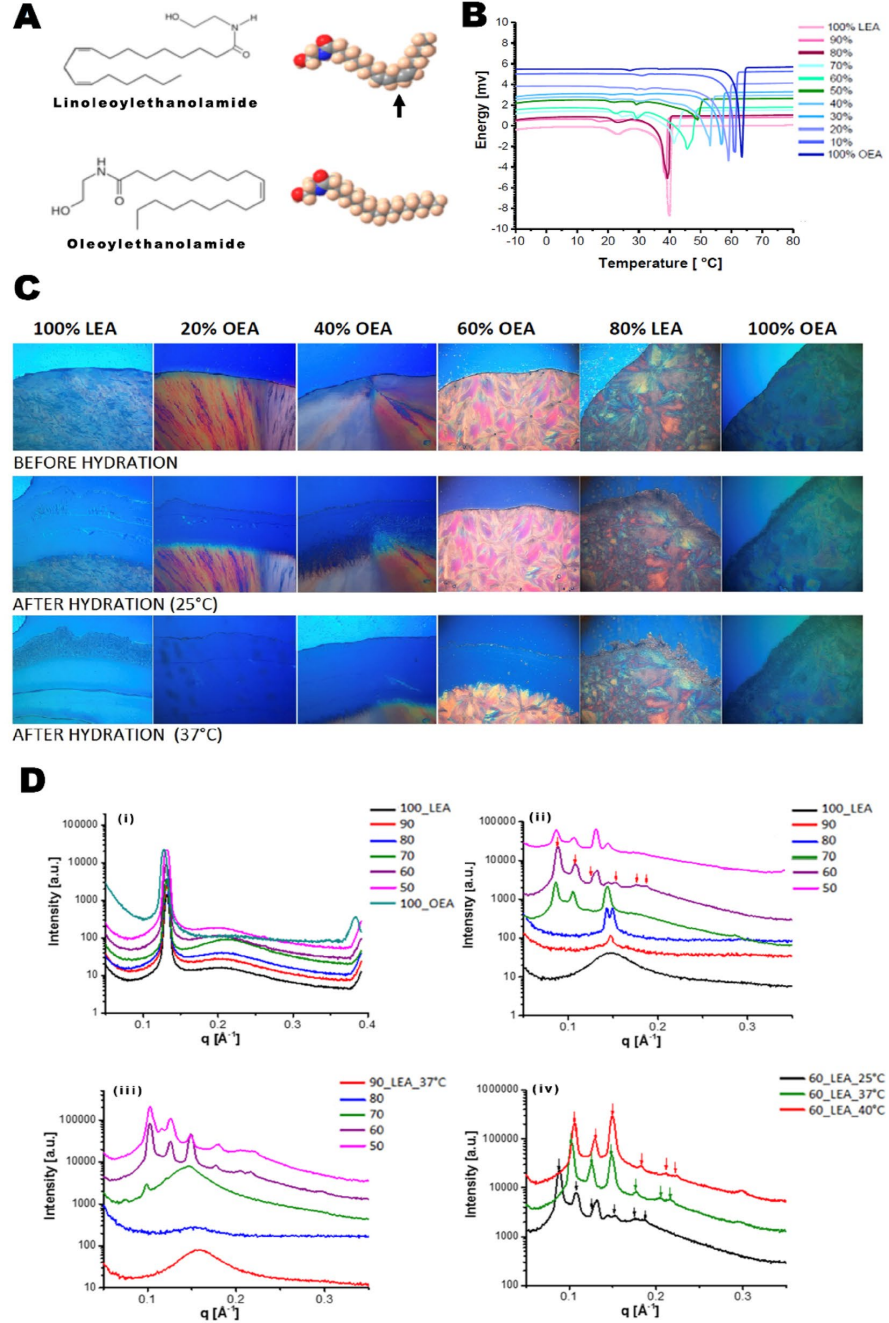
(**A**) Chemical structure and space filling molecular model of endocannabinoid lipids OEA and LEA. (**B**) Differential scanning calorimetry showing the melting behaviour of amphiphile mixtures composed of LEA and OEA, representing one single melting point of each mixture. (**C**) Optical microscopy of pure monoethanolamide lipids LEA and OEA; amphiphile mixtures at varying LEA to OEA ratios. Images acquired at 25 °C and 37 °C before and after hydration from a fixed position (magnification × 100). Different mesophases are observed from pure water to pure amphiphile. Polymorphic changes in the mesophases from cubic to a more lamellar phases were observed as the OEA to LEA ratio increased. (**D**) SAXS profiles of; (i) bulk and; (ii–iv) lyotropic mesophases of mixed LEA/OEA in excess water (70 wt%). The lyotropic mesophases were equilibrated for 48 h and analysed at 25 °C (ii) and 37 °C (iii). Lyotropic mesophases of 60% LEA at various temperatures (iv), show a cubic mesophase with Pn3m space group. The scattering vector q = (4π/λ) sin(θ/2), where λ is the X-ray wavelength and θ is the scattering angle.

Examination of the thermal behaviour of the pure LEO and OEA indicated that the crystal-isotropic melt for the mixed amphiphiles showed a systematic monotonic shift in the transition temperatures (Fig. 1B), which was dependent on the degree of unsaturation in the hydrocarbon chain (Fig. 1A). Insight into the lyotropic phase behaviour of NAE self-assembly was examined by the water penetration scan technique and using POM (Fig. 1C). By mixing OEA and LEA in different ratios and regulating the percentage of unsaturation in the lipid mix, stable NAE NPs were formed. The NPs were characterized using DLS, cryo-TEM, and SAXS. The average hydrodynamic radii of the dispersed NP’s measured by DLS (Supplementary Fig. 2A) sized the NP at 170 d.nm with a polydispersivity index (PDI) of 0.124. Cryo-TEM images (Supplementary Fig. 2B) showed that colloidal dispersions of 40% OEA adopted a lamellar structure consistent with the formation of liposomes, and the average size of the imaged particles agreed with the data obtained by the DLS.

The SAXS bulk and lyotropic mesophases of pure mixed LEA/OEA are shown in Fig. 1D (i). All the amphiphilic mixtures examined showed a relatively sharp peak in addition to a broad peak. The sharp peak of 100% LEA at ~ 0.132 Å^−1^ is indicative of an ordered lamellar mesophase. As the percentage of OEA was increased, there was a shift in the sharp peak to lower q values indicating a slight increase in the lattice parameter. The lyotropic mesophases of the hydrated samples at 70 wt% water (excess water) at 25 °C are shown in Fig. 1Dii. The SAXS pattern of mixtures ranging from 100% LEA to 50% LEA showed the formation of varying ordered nanostructures. The shift to lower q value from those observed in the pure amphiphile is indicative of a swollen L2 mesophase with a weakly ordered nanostructure. As the LEA ratio was increased, the molten mesophase changed slightly and a sharp peak appeared at 0.147 Å^−1^. At 70% LEA a mixed cubic mesophase, along with a crystalline lamellar peak at 0.143 Å^−1^ was observed. At 60% LEA, the cubic mesophase became more dominant however transformed to a mixed cubic and lamellar mesophase at 50% LEA. The lyotropic mesophases of the hydrated mixtures was assessed at 37 °C (Fig. 1Diii). Raising the temperature of the hydrated samples to physiological temperature resulted in more molten L2 mesophase for mixtures containing 90–70% LEA. However, at 60% LEA, a mono cubic mesophase with the Pn3m symmetry was developed. At 50% LEA, there is a shift from sole cubic mesophases (as seen in 60% LEA), with both cubic mesophase mixed with a lamellar liquid crystalline mesophase observed. From this detailed analysis, 60% LEA 40% OEA mixture showed a sole cubic mesophase of Pn3m symmetry, which was stable up to physiological temperature (Fig. 1D(iv))^22,23^. When stabilized with PEG2000 conjugated with synovia-targeted peptide HAP-1, 60% LEA 40% OEA system was selected for further study including detailed biological evaluation. 1D SAXS analysis on the stabilized, targeted NPs formed at 40%OEA/60%LEA is shown in Supplementary Fig. 2C.

### NP functional studies

NP in vitro cell cytotoxicity was assessed by colorimetric assay WST-1 as shown in Supplementary Fig. 3A. The LC50 was 40 µg/mL after a 24 h incubation period and NPs were subsequently used at 30 mg/mL concentration.

**Figure 2:**
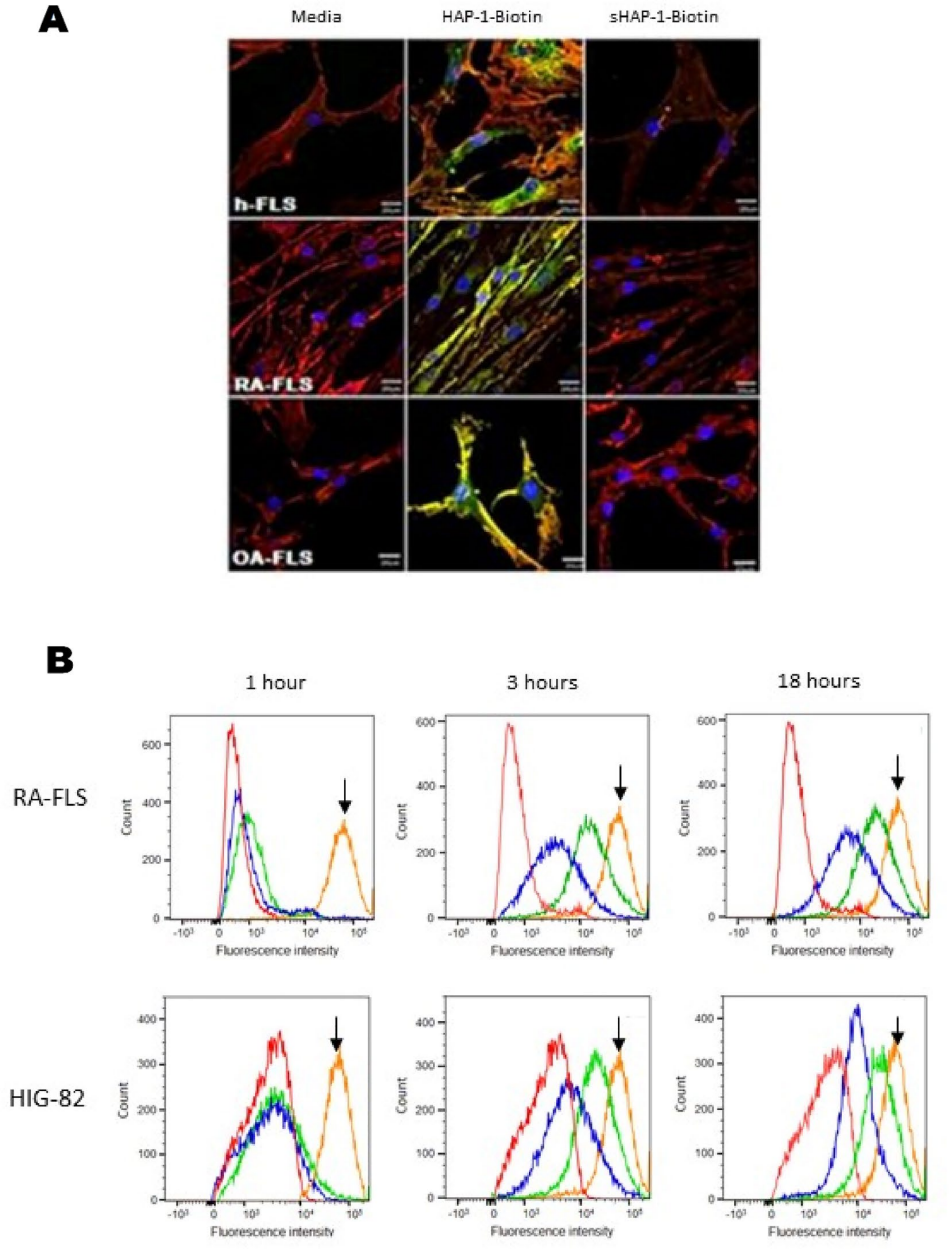
(**A**) Confocal microscopy of HAP-1-binding to human (h)-FLS, RA-FLS and OA-FLS cells. Cells at 37 °C were incubated with media, HAP-1-biotin, or sHAP-1-biotin followed by streptavidin-FITC. Actin is labelled with TRITC-phalloidin (red), HAP-1-biotin with streptavidin-FITC (green), and nuclei with DAPI (blue). Scale bars represent 100 μm. Positive binding was present for HAP-1-biotin treated HIG-82 and all h-FLS cell types. The bin ding pattern of HAP-1-biotin appeared to be consistent across the RA and OA h-FLS cell types with positive staining present on both the surface (yellow) and cytoplasmic internalization (green). Cells incubated with either media or sHAP-1-biotin showed no green FITC-stain demonstrating the binding specificity of the HAP-1 sequence. (**B**) Flow cytometry histograms illustrating uptake of NP_non-targeted_ and NP_HAP-1_ following incubation for 1, 3 and 18 h at 37 °C with RA-FLS or HIG-82 cells. Autofluorescence is shown as red, incubation with NP_non-targeted_ as blue, incubation with NP_HAP-1_ green, and incubation with free DiD dye (orange, shown as arrow). The experiments were repeated three times and the histogram is the average.

**Figure 3:**
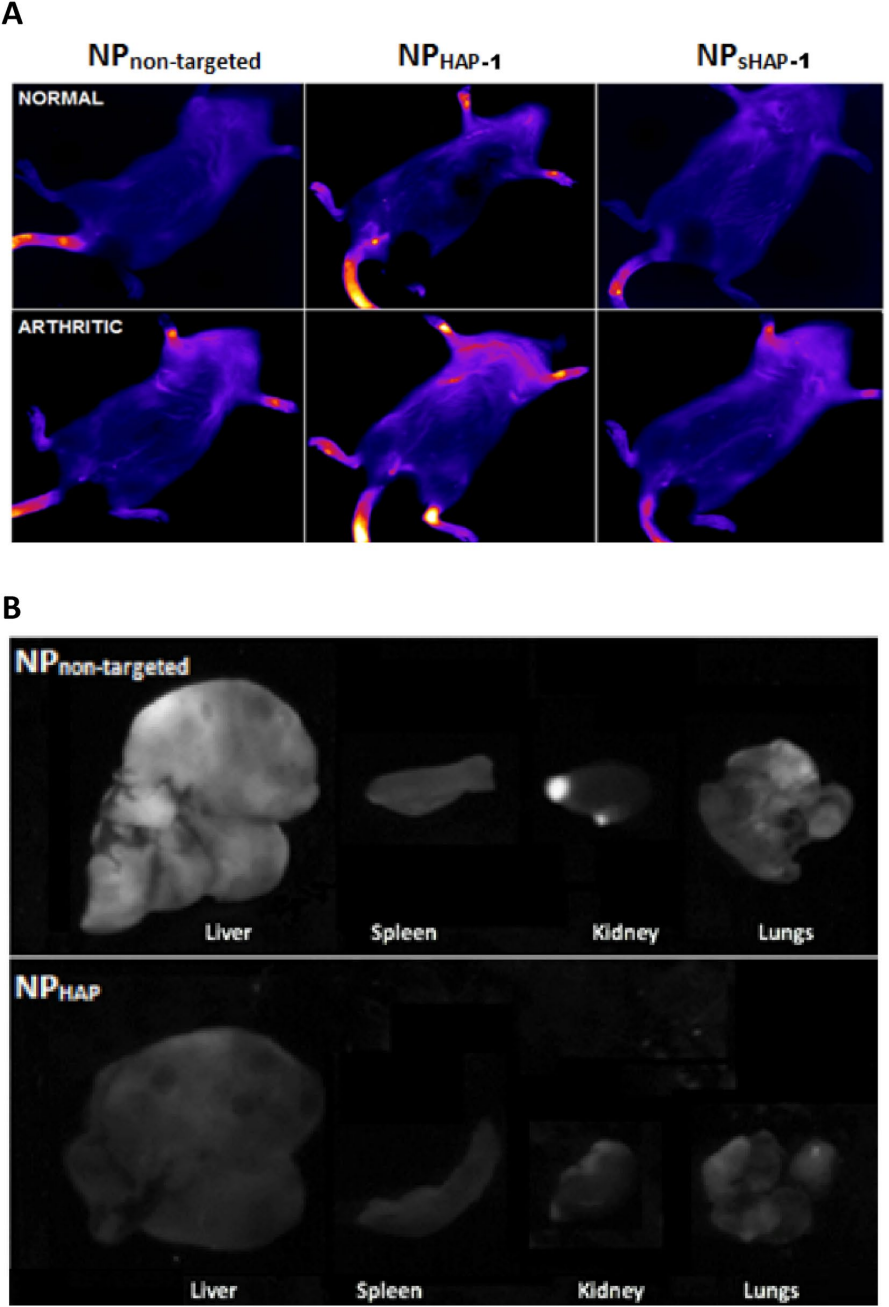
In vivo NP localization in normal and arthritic rats using near infrared imaging (NIR). (**A**) Localization of NP_non-targeted_, NP_HAP-1_ and NP_sHAP-1_ 24 h post-injection. No specific accumulation was seen in normal rats treated with NP_non-targeted_ and NP_sHAP-1_. In arthritic rats, slight accumulation to the inflamed joints was observed for NP_non-targeted_ and NP_sHAP-1_. For NP_HAP1_, localization to the joints was observed in both normal and arthritic rats. (**B**) Ex vivo imaging of liver, spleen, kidney, and lungs extracted from NP_non-targeted_, NP_HAP-1_ rats.

**Figure 4:**
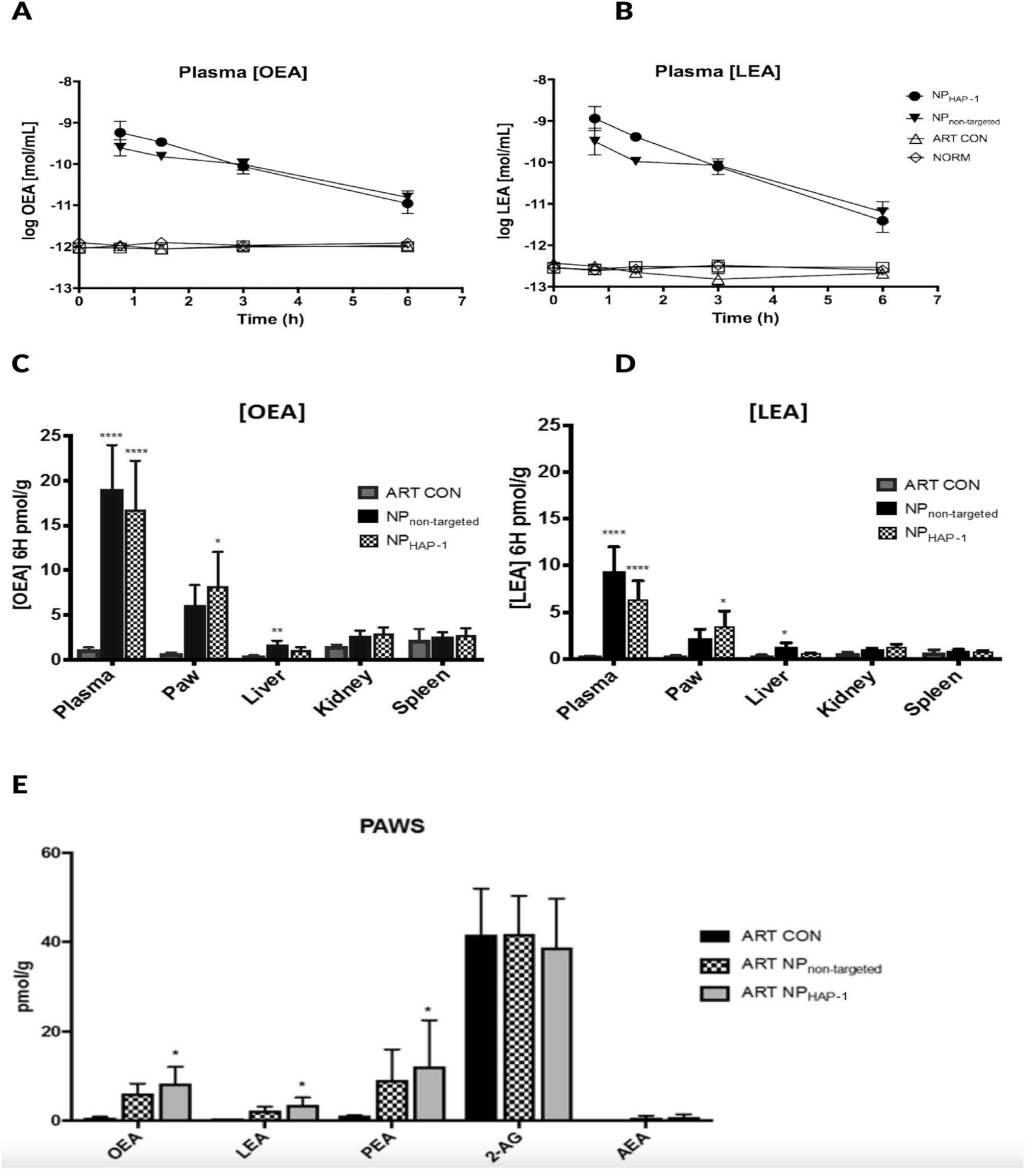
Plasma concentrations and biodistribution of OEA and LEA in normal rats (NORM), arthritic control rats (ART CON) and arthritic rats treated with NP_non-targeted_ (ART NP_non-targeted_) and NP_HAP-1_ (ART NP_HAP-1_). NPs were intravenously administered, and blood collected at 0, 45 min, 1.5, 3 and 6 h. After 6 h, organs and paws were harvested and analyzed on MS. Plasma concentration of; (**A**) OEA and (**B**) LEA concentration expressed as mol/mL (mean ± S.D., n = 5). Organ and paw concentrations of (**C**) OEA and (**D**) LEA expressed in pmol/g. (mean ± S.D., n = 5). *p < 0.05, ****p < 0.0001 vs ART-CON, Uncorrected Fisher’s least significance difference, two-way ANOVA. Significant accumulation to the paw was observed in NP_HAP-1_ treated rats. (**E**) Regulation of endogenous endocannabinoid OEA, LEA, PEA, 2-AG and AEA. Data expressed as pmol/g (mean ± S.D., n = 8). (*p < 0.05 vs ART CON, analysis using one-way ANOVA). Significant concentrations of OEA, LEA in ART NP_HAP-1_ were correlated to increases in PEA.

- *In-vitro *The staining pattern for HAP-1 biotin was similar in HIG-82, RA-FLS and OA-FLS cell types (Fig. 2A). To determine in vitro homing peptide HAP-1 binding and internalization into FLS cells, biotin-conjugated HAP-1 and controls were added to HIG-82 (rabbit), human (h) osteoarthritic and rheumatoid arthritic (OA and RA, respectively) derived FLS followed by avidin-FITC staining. Representative confocal images of HAP-1 binding to HIG-82 cells are shown in Supplementary Fig. 3B. A similar staining pattern was noted with RA-FLS and OA-FLS cell types (Fig. 2B). To evaluate the FLS binding and uptake of NPs_,_ fluorescent labelled NP_non-targeted_ and NP_HAP-1_ were incubated with h-FLS cells at 37 °C for 1–3 h and signal fluorescence assessed using flow cytometry. As shown in Fig. 2B there was no detectable free dye or leakage recorded for NP_non-targeted_ and NP_HAP-1_ following 1 h incubation with HIG-82/h-FLS cells. Following 3 h incubation (Fig. 2B), NP_HAP-1_ was internalized more efficiently than NP_non-targeted_ and the cell fluorescence peak shifted higher intensity, with the percentage of cells that took up detectable dye recorded as 69.1% for NP_HAP-1_ and 33.2% for NP_non-targeted_. Increasing NP contact to 18 h (iii, vi) improved uptake of both NP_HAP_ and NP_non-targeted_ to 74.5% and 44.8%, respectively, when compared to 3 h exposure. Cell fluorescence at 4 °C and 37 °C for 3 h is shown in Supplementary Fig. 3C. At 4 °C there was no fluorescence from cells incubated with either DiD labelled NP_non-targeted_ or DiD labelled NP_HAP-1_ suggesting that the encapsulated DiD dye was retained within the NP. Conjugation of HAP-1 to the NPs facilitated the preferential uptake of NP_HAP-1_ by h-FLS cells when compared to NP_non-targeted_ in vitro suggesting a receptor-mediated process.
- *In-vivo* Fluorescent-tagged NP_non-targeted_, NP_sHAP-1_, and NP_HAP-1_ were injected into normal and arthritic rats and their localization tracked using a NIR imager. NP accumulation was measured as fluorescence intensity captured at 1, 3, 24 and 48 h for 10 min. Figure 3A shows NIR images taken at 24 h post injection. In contrast to the NP_non-targeted_ and NP_sHAP-1_ groups, normal rats injected with the targeted NP_HAP-1_ localized to joints (34.37 ± 2.08 signal units). Signal at these joints was 73.6% higher than that observed in NP_non-targeted_ (19.79 ± 6.04 signal units) normal rats. In arthritic rats, localization of NP_HAP-1_ to the inflamed joints increased 58.2% (54.37 ± 13.95 signal units) compared to NP_HAP-1_ treated normal rats; and 69.3% higher than NP_non-targeted_ arthritic rats. Following whole-animal imaging at 24 h, the rats were sacrificed and the major internal organs; spleen, liver, kidneys, heart, and lungs were harvested for NIR imaging (Fig. 3B). Minimal fluorescence was observed in the heart, kidneys, spleen, and lungs. In contrast, strong signal was noted in liver rather than other sites suggesting greater clearance of the NPs at this site. Fluorescence of the liver from the NP_non-targeted_ group was significantly higher when compared to the liver of the NP_HAP-1_ injected group.
- *Biodistribution of targeted and non-targeted NP* Blood plasma levels of OEA, LEA, endogenous NAE levels, and their entourage compounds, 2-arachidonyl glycerol (2-AG), N-arachidonoyl-ethanolamide (AEA), palmitoylethanolamide (PEA) were measured in normal rats (NORM), untreated arthritic rats (ART CON), and NP treated arthritic rats. Figure 4 shows the plasma concentrations of; (A) OEA and (B) LEA over 6 h. The plasma half-life for NP_non-targeted_ was 0.16 h (LEA), 0.20 h (OEA) and the half-life of NP_HAP-1_ 0.31 h (LEA) and 0.48 h (OEA). The distribution of NP OEA and LEA lipids in solid organs was determined at 6 h post NP administration (Fig. 4C,D), measured against baseline levels of control arthritic rats. While concentrations of NP_non-targeted_ were high in the liver, only small amounts of OEA (0.86 ± 0.09 pmol/g) and LEA (0.59 ± 0.10 pmol/g) were recorded in the liver for NP_HAP-1_ treated groups. These concentrations were much lower than that observed for NP_non-targeted_, and were in agreement with the lower fluorescence noted in the liver from NP_HAP-1_ treated rats imaged by NIR. Similarly, NP localization to the the kidneys [(OEA 2.97 ± 1.30 pmol/g) and LEA (1.29 ± 0.57 pmol/g)] and spleen [(2.79 ± 1.58 pmol/g) and LEA (0.79 ± 0.219 pmol/g)] was minimal and comparable to that seen in NP_non-targeted_.

In the NP_HAP-1_ arthritic paws, there were significantly higher levels of OEA (8.26 pmol/g of tissue, p < 0.05) and LEA (4.16 pmol/g of tissue, p < 0.05) compared to NP_non-targeted_ group. Tissue:plasma ratio of OEA and LEA NP_non-targeted_ and NP_HAP-1_ treated arthritic rats is shown in Supplementary Materials and Methods (Supplementary Fig. 4C,D, respectively). The data agree with the localization results obtained by NIR, which showed a significant uptake of NP_HAP-1_ in comparison to NP_non-targeted_ in arthritic joints.

In addition, to OEA and LEA measurements, local joint endocannabinoids PEA, 2-AG and AEA were measured (Fig. 4E). Significant concentrations of OEA and LEA in the NP_HAP-1_ treated group were correlated with significant increases in PEA (12.26 ± 10.27 pmol/g, p-value 0.0393). Elevated levels of PEA were also recorded for NP_non-targeted_ (9.13 ± 6.85 pmol/g, p-value 0.0409) treated rats, which were not significant when compared to baseline levels. Similarly, levels of AEA were slightly increased in both NP_HAP-1_ (0.78 ± 0.65 pmol/g, p-value 0.1784) and NP_non-targeted_ (0.64 ± 0.48 pmol/g, p-value 0.0689) treated rats when compared to ART CON (0.07 ± 0.01 pmol/g), however these levels were not significant. In contrast to PEA, endogenous levels of 2-AG remained relatively unaffected in both NP_HAP-1_ (38.84 ± 10.86 pmol/g) and NP_non-targeted_ (41.92 ± 18.91 pmol/g) treated rats when compared to ART CON (41.68 ± 10.32 pmol/g).

### NP cytokine effects in-vitro and in-vivo

**Figure 5:**
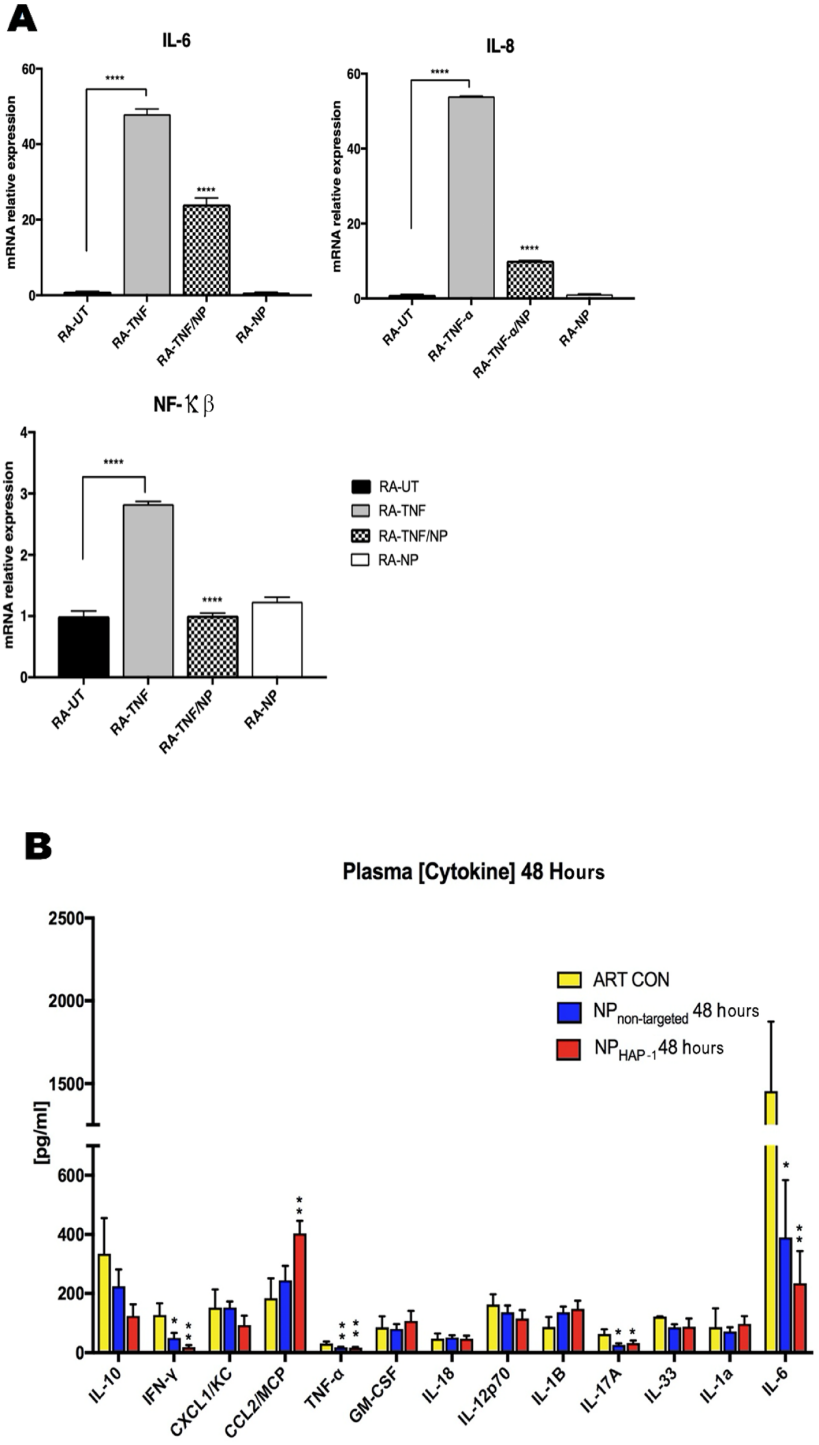
NP effect on cytokine production. (**A**) In vitro effects showing NP suppresses pro-inflammatory upregulation of NF-κβ, IL-6 and IL-8 mRNA in RA-FLS cells. Cultured RA-FLS were stimulated with TNF-α alone (25 ng/mL) or TNF-α plus NP (30 µg/mL) for 24 h. Expression levels of mRNA were assayed by quantitative real-time RT-PCR. The mRNA levels of each gene were standardized against GAPDH levels. Data are expressed as the mean (S.D.) (n = 4). *p < 0.05; **p < 0.01, ***p < 0.001, ****p < 0.0001 vs TNF-α alone. NP significantly inhibited TNF-α stimulated production of IL-6, IL- 8 and NF-κβ. (**B**) In vivo cytokine effects of NP in an arthritis model of inflammation. Plasma concentrations of cytokines in non-treated (ART CON), NP_non-targeted_ and NP_HAP-1_ treated arthritic rats were measured. Arthritic rats were treated with NP intravenously administered once daily, for two days and blood collected 48 h after the initial injection. Low plasma concentrations of pro- inflammatory cytokines IFN-γ, IL-6 and IL-17A were observed in both the NP_non-targeted_ and NP_HAP-1_ treated rats. Data expressed as the mean + S.D. (n = 6). *p < 0.05; **p < 0.01 against ART-CON.

- *In-vitro* Incubation of cultured RA-FLS with recombinant TNF-α (10 ng/mL) induced marked increase in the gene transcription of NF-κβ, IL-6 and IL-8 (Fig. 5A) by a fold change increase of NF-κβ 2.832 (p < 0.0001, n = 3), IL-6 47 (p < 0.0001, n = 3) and IL-8 53 (p < 0.0001, n = 3), respectively. In contrast, TNF-α stimulated cells treated with NPs significantly decreased expression of NF-κb 0.64 (p < 0.0001, n = 3), IL-6, 0.5-fold (p < 0.0001, n = 3) and IL-8 0.8-fold (p < 0.0001, n = 3), respectively. These results are consistent with data observed in RNA-seq analysis.
- *In-vivo* To investigate the in vivo cytokine effects of NP, arthritic rats were injected with NP intravenously once daily, for 2 days, and the following serum cytokines and chemokines—IL-1α, IL-1α, IL-2, IL-4, IL-6, IL-10, GM-CSF, IFN-γ, and TNF-α measured. The control arthritic group received PEG/PBS (vehicle diluent) intravenously in similar volumes. As shown in Fig. 5B, NP_non-targeted_ treatment suppressed pro-inflammatory cytokines IL-6 by 73.3% when compared to arthritis control (p-value: 0.03); TNF-α by 42% p-value: 0.002; IL-17A by 70.6%, p-value: 0.05; and IFN-γ by 61.3%, p-value: 0.03) pg/mL. Similar cytokine inhibition was observed with NP_HAP-1_, which decreased IL-6 by 83.9%, p-value; 0.01, TNF-α by 47.4% p-value; 0.003), IL-17A by 53.8%, p-value; 0.02) and IFN-γ by 85.3% p-value; 0.003. The suppression of IFN-γ was significantly more pronounced in NP_HAP-1_. NP_HAP-1_ treated rats had significantly increased circulating concentrations of monocyte chemoattractant protein-1/chemokine ligand 2 (MCP/CCL2) (403.26 ± 42.52 pg/mL vs ART-CON 184.69 ± 66.67 pg/mL), which was not statistically significant with NP_non-targeted_ treated rats (244.82 ± 48.87 pg/mL). CCL2 which was increased, significantly reduces insulin-stimulated glucose uptake in myocytes and may play an important metabolic role in inflammation. RNA-seq was subsequently performed to examine the metabolic effects and other pathways that might be affected by NPs. NP treatment significantly reduced the concentration of several pro-inflammatory cytokine serum levels in arthritic rats.

### RNA sequencing (RNA-SEQ)

Raw read and quality metrics of RNA-seq data that were aligned to the GRCh37/hg19 reference genome using STAR are shown in Supplementary Table 1. A heat map of the top differentially expressed (DE) genes in RA-FLS treated groups is shown in Supplementary Fig. 6. Log fold expression is represented on a z-score color scale, where red denotes highly expressed genes and blue denotes low expression. Assessment of the top DE genes in RA-TNF showed the acquisition of genes associated with inflammation and joint erosion. As shown, the upregulated chemokines (CXCL3,5,6,8,10; CCL5, CCL20, CX3CL1, CXCR4) are often elevated in RA serum and are important in the recruitment of inflammatory mediators and angiogenesis. In addition to the chemokines, a pronounced recruitment of genes involved in inflammatory signalling (TRPA1, IL23A, LBP, GBP4, TREM1, HLA-F) and bone remodelling (IBSP, BST2, ASPN) was also observed (Supplementary Table 2). These include genes involved in osteoclastogenesis (CXCL8, CCL20, IL-23) and breakdown of extracellular matrix proteins (MMP-3, CRTAC1), all of which collectively contribute to inflammation and degenerative joint function associated with RA. By contrast, assessment of the top DE genes in TNF/NP showed a significant reduction in inflammatory and bone remodelling genes, previously upregulated in RA-TNF. In addition to reduction of these genes, NP treatment promoted the acquisition of genes associated with homeostasis and inflammation resolution (SERPINB2, Hsp70; HSPA6, HSPA7, TFPI2, IL1RN). In contrast, genes CXCLorf48, MMP-3 and CXCL3,6 appeared to be largely unaffected by NP treatment, while CXCL5,8 was slightly upregulated. Overall, assessment of top DE genes in each group highlights an immunological shift from highly pro-inflammatory in an acute inflammatory environment, mediated by NP treatment.

**Figure 6:**
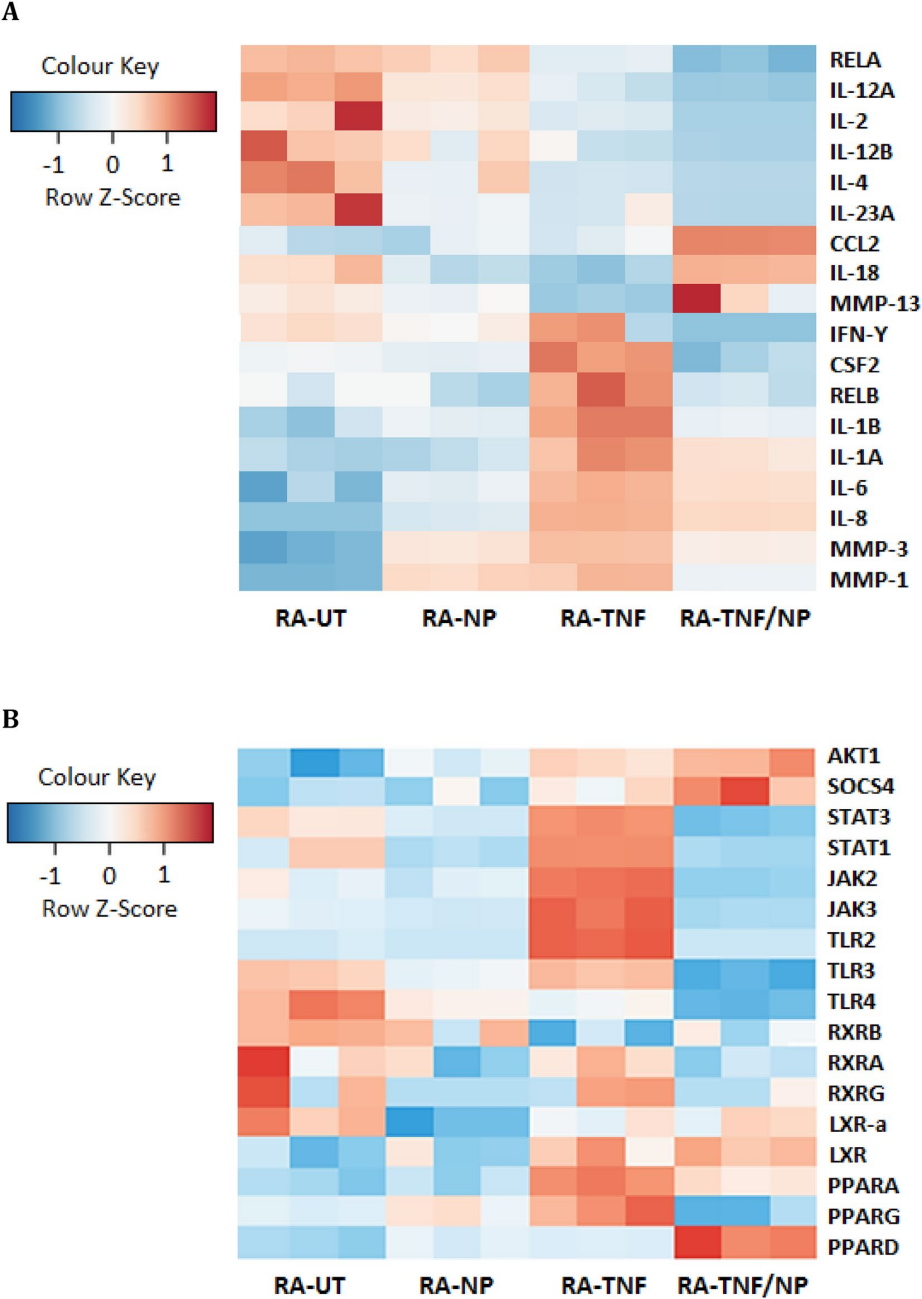
Heat map of differential expressed genes prominent in arthritis progression in RA-FLS cells. Groups included untreated RA-FLS cells (RA-UT), TNF-α stimulated cells (RA-TNF), NP and TNF-α stimulated cells (RA-TNF/NP), and NP treated cells (RA-NP). The normalized RNA-seq data is in log2 scale, where red is highly expressed genes and blue is low expression. To be included in the heat map, genes were required to have at least 1000 counts (reads), totalled over all samples, where the standard deviation of log2 expression differences had to exceed two. (**A**) Heat map of DE candidate genes prominent in arthritis progression in RA-FLS cells. The heatmap highlight anti-inflammatory effects of NP in TNF-α induced pro-inflammatory cytokine expression. Abbreviations: NF-κβ subunits (RELB, RELA); Interferon, (IL-(12A, 12, 12B, 4, 23A, 18, 1B, 1A, 6, 8)); Matrix metalloproteinases (MMP-[1, 3, 13]); chemokine ligand 2 (CCL2); colony-stimulating factor 2 (CSF2). (**B**) Heat map of DE candidate signalling genes. The heatmap highlight shift in gene expression following NP treatment. Abbreviations: Serine/threonine-protein kinase (AKT1); Suppressor of cytokine signalling 4 (SOCS4); Janus kinase (JAK-[2, 3]); Signal transducers and activators of transcription (STAT-[1, 3]); Toll like receptor (TLR-[2–4]); Liver X receptor (LXR-[α]); Retinoid X receptor (RXR); Peroxisome proliferator- activated receptors (PPAR [A,G,D]-[α, ɣ, δ]).

- *Regulation of candidate pro-inflammatory mRNA* The expression of key mediators in arthritis is shown as a heat map in Fig. 6. These candidate mRNAs were specifically selected and screened due to their importance in inflammatory and disease progression in RA. RA-TNF/NP and RA-TNF groups were compared and examined to assess NP regulation of key inflammatory genes in a TNF-α induced acute inflammatory response in vitro. As shown in Fig. 6A, highly expressed pro-inflammatory cytokines IL-1β, IL-1α, IL-6, IL-8, TGFβ and IFN-Ƴ in RA-TNF cells, were shown to be down regulated in RA-TNF/NP groups. Similarly, TNF-α induced upregulation of MMP-1 and MMP-3 were also shown to be suppressed following NP incubation in RA-TNF/NP groups. NF-kβ dimers, REL and RELA appeared largely unaffected by NP incubation, while RELB was suppressed in RA-TNF/NP. In contrast, CCL2/MCP-1 genes were shown to be more highly expressed in RA-TNF/NP when compared to RA-TNF alone. The up-regulation of these genes was not shared by the RA-NP group, and was specific to and dependent on the presence of TNF-α. Similar, to RA-TNF/NP, highly expressed inflammatory cytokines in RA-UT were downregulated following NP treatment in RA-NP, in particular IL-4, IL-12B, IL-13, IL-2, IFN-Ƴ and CCL2. In contrast, expression of TGFβ and collagenases MMP-1 and MMP-3, while downregulated in RA-TNF/NP, were upregulated in RA-NP when compared to RA-UT.
  Figure 6B illustrates the regulation of candidate mRNA genes involved in inflammatory signalling, (abbreviations are supplied in figure legend). As expected, clustering based on the expression pattern of the chosen signalling genes resulted in a clear separation between RA-TNF and RA-UT, as indicated by opposite color scoring in the z-score. In RA-TNF, high expression of JAK, STAT, PPAR and TLR2, and to a lesser degree AKT1 were found to be upregulated when compared to RA-UT. The increase in signalling genes following TNF-α are consistent with the pro-inflammatory stimulatory effects of the cytokine. By contrast, TLR3 remained largely unaffected, while TLR4 was downregulated. For NP-treated cells, there was a significant shift in gene regulation, with highly expressed genes in RA-TNF oppositely expressed in RA-TNF/NP. In particular, TLR, STAT, RXR and PPAR-Ƴ genes were significantly downregulated, while PPAR-δ, SOCS4, AKT1 and LXR genes were significantly upregulated. This suggests that anti-inflammatory effects are mediated by regulation of these genes. Similarly, comparison of RA-NP to RA-UT showed a shift in gene regulation consistent with that seen in RA-TNF/NP when compared to RA-TNF, however to a lesser degree. This suggests that effects are mediated by a similar pathway. In particular, TLR and RXR genes, which were highly regulated in RA-UT, were downregulated in RA-NP.
- *Top up-stream regulators in RA-FLS treated groups* In the RA-TNF group, the top five regulators were TNF (p-value 4.10 × 10^–48^), IFN-γ (p-value 5.97 × 10^–36^), REL-associated protein involved in NF-κβ heterodimer formation (RELA; p-value 1.16 × 10^–28^), IL-1α (p-value 5.73 × 10^–25^) and NF-κβ (p-value 3.59 × 10^–23^) when compared to RA-UT cells alone (Supplementary Table 3A). These regulators are all heavily involved in promoting inflammation and known to be ‘activated’ following TNF-α stimulation of the RA cells. By contrast, the TNF-α stimulated cells which were co-incubated with NP had an opposite regulatory effect. The top five regulators for the RA-TNF/NP groups were TNF (p-value 1.70 × 10^–54^), IFN-Ƴ (p-value 2.64 × 10^–48^), IL1B (p-value 3.29 × 10^–35^), lipopolysaccharide (p-value 56.52 × 10^–34^) and IFN-α (p-value 2.87 × 10^–31^), when compared to RA-TNF. While these regulators were shared between the RA-TNF groups, they were shown to be ‘inhibitory’ following NP incubation in RA-TNF/NP. Inhibition of these key cytokines involved in inflammatory regulation would help contribute to reduced secretion of pro-inflammatory cytokine observed in a stimulated in vitro model of acute inflammation (Supplementary Table 3B). The top ten differentially expressed genes when comparing TNF-α treated RA-FLS cells and NP treated cells (RA-TNF/NP) are shown in Table 1.
- *Network and pathway analyses of differentially expressed genes* Pathways analysis of differentially expressed genes was performed using Ingenuity Pathway Analysis software (Supplementary Fig. 7A–C). Genes significantly expressed following stimulation with TNF-α led to enrichment of cytokine-rich ontologies. The top canonical pathways induced in the TNF-α stimulated RA-FLS cells were hepatic fibrosis/hepatic stellate cell activation (p-value 6.85 × 10^–11^, overlap 21.9%), granulocyte adhesion and diapedesis (p-value 7.65 × 10^–10^, overlap 21.7%), dendritic cell maturation (p-value 1.34 × 10^–09^, overlap 20.5%), agranulocyte adhesion and diapedesis (p-value 4.08 × 10^–09^, overlap 20.5%) and neuroinflammation signaling pathway (p-value 2.25 × 10^–08^, overlap 16.3%), Supplementary Table 4A. Similarly, the top canonical pathways in the RA-TNF/NP group, shown in Supplementary Table 4B, were granulocyte adhesion and diapedesis (p-value 3.32 × 10^–11^, overlap 21.1%), hepatic fibrosis /hepatic stellate cell activation (3.36 × 10^–11^, overlap 20.2%), agranulocyte adhesion and diapedesis (2.93 × 10^–9^, overlap 18.8%), role of macrophages, fibroblasts, and endothelial cells in rheumatoid arthritis (8.23 × 10^–8^, overlap 14.2%). LXR/RXR activation (1.56 × 10^–09^, overlap 22.3%) was negatively regulated.

**Table 1:** Top 10 differentially expressed genes based on comparison of RNA-seq data between TNF-α and NP treated cells (RA-TNF/NP), and TNF-α treated (RA-TNF) RA-FLS cells.

Gene	Description	Log2 FC	Padj	Type(s)
**Top 10 top-regulated genes**				
SERPINB2	Serpin family B member 2	10.458	5.3E−118	Other
MRGPRX3	MAS related GPR family member X3	-9.952	1.03E−15	G-protein coupled receptor
TLR2	Toll like receptor 2	-9.64	1.1E−14	Transmembrane receptor
HSPA6	Heat shock protein family A (Hsp70) member 6	9.603	1.32E−19	Enzyme
IL36B	Interleukin 36 beta	9.366	3.25E−14	Cytokine
HSPA7	Heat shock protein family A (Hsp70) member 7	9.241	1.78E−13	Other
CX3CL1	C-X3-C motif chemokine ligand 1	-9.204	2.14E−292	Cytokine
FOXI1	Forkhead box I1	-9.128	4.9E−13	Transcription regulator
NOS2	Nitric oxide synthase 2	-8.597	1.78E−11	Enzyme
ANO9	Anoctamin 9	-8.584	1.35E−15	Ion channel

## Discussion

This study highlights a novel delivery system and a promising use of endocannabinoid-targeted NPs as a therapeutic tool for treating inflammation, using as an example a robust adjuvant-induced arthritic rat model. The results of this study are not restricted to arthritis since the homing molecules on the NPs can be altered to target any tissue of interest. Treatment of other medical conditions may include, for example, epilepsy (brain tissue), malignancies (melanoma, lymphoma), psoriasis (skin) and interstitial fibrosis (lung). Conjugation of synovium-targeting peptide, HAP-1, to the surface of the NPs resulted in specific binding and greater uptake of NPs to human fibroblast-like cells, demonstrating the ability to actively target therapeutics to the inflamed synovium in vivo. In addition to active targeting, this study demonstrates the encouraging anti-inflammatory effect NPs exert at the site of localization, due to the release of endocannabinoids from the shell structure.

Consideration of the NP size, morphology and nanostructure using several techniques such as Nanosizer, cryo-TEM and SAXS supports the formation of spherical particles of spongosomes and liposomes. These structures are inconsistent with the highly ordered 3D nanostructures of the cubic phase, as observed by SAXS analyses of the lyotropic phases in bulk studies (shown in Fig. 1D(iv)). The change in the nanostructures of the NPs from those observed in the bulk lyotropic phase is likely to be due to the incorporation of PEGylated molecules with large hydrophilic head groups. Inclusion of Ole-PEG2000-OH and its combination with Ole-PEG2000-HAP-1 changed the packing curvature of the membrane. Therefore, this inclusion likely contributed to changes in the curvature of the membrane of the 3D cubic phase self-assembled structure and transformed the self-assembly of the main framework of the NP made from 60% LEA/40% OEA. This would have resulted in more flattened liposomal particles or less ordered spongosomes. These results indirectly imply the inclusion of PEGylated-Ole and its combination with PEG-2000-HAP for non-targeted or targeted NPs. Using lower percentages of stabilizers could retain the structure of the bulk mesophases^16^.

Testing the NPs in vitro and in vivo shows that conjugation of synovium-targeting peptide, HAP-1, to the surface of the NPs is feasible and results in specific binding with greater uptake of NPs to FLS. For HAP-1 this appears to be a receptor-mediated process. Biodistribution of targeted and non-targeted NPs indicates that the release of NP membrane elements is greater at the sites of localization. This enhances local cannabinoid levels and can regulate endogenous endocannabinoid quantities and cytokine production, reducing inflammation. Using RNA-SEQ, the anti-inflammatory effects appear to be mediated by inhibition of cytokine pathways, TLR, JAK-STAT, and PPAR signalling, as well as the regulation of transcriptional factors at sites of inflammation. These findings support previous studies showing that OEA and LEA have inherent anti-inflammatory and analgesic properties^9,15,24^. The ability of the NPs to suppress key cytokines IL-6, IFN-Y, TNF-α, IL-17A, NF-κβ, and regulators of key canonical pathways such as granulocyte adhesion and diapedesis, macrophage activation, fibroblast and endothelial cell function, LXR (liver X receptors)/RXR (retinoid X receptor α) activation, and neuro-inflammation suggests the potential application in other areas of disease. The ability of LXR/RXR to integrate metabolic and inflammatory signalling makes them particularly attractive targets for intervention in human metabolic disease and neurological conditions such as Alzheimer’s disease^25^. The regulation of the key hepatic fibrosis canonical pathway suggests the potential of NPs as therapeutic agents for liver fibrosis, interstitial lung disease, scleroderma and other fibrosing conditions. In vivo, injection of OEA significantly attenuates the progress of liver fibrosis by blocking the activation of hepatic stellate cells^26^. The regulation of granulocyte adhesion and diapedesis pathway suggests NPs may have therapeutic application in allergic inflammation by regulating pro-inflammatory mediators, including vasoactive amines, cysteinyl leukotrienes and cytokines. The neuro-inflammatory canonical pathway was shown to be regulated by the NPs. In a separate study, peripheral administration of OEA was shown to cross the blood–brain barrier^27^ and activate PPARα, preventing LPS-induced increases in cortical TNF-α mRNA levels and reduced NF-κβ activation, the expression of inducible nitric oxide synthase (iNOS) and cyclooxygenase-2 (COX-2), accumulation of nitrogen dioxide (NO_2_), and lipid peroxidation in the frontal cortex^28^. Given the importance of neuro-inflammation in the physiopathology of neuropsychiatric diseases, the results suggest that OEA/LEA-based NPs might help delay the onset of neurodegenerative and neuropsychiatric diseases by reducing the insults to brain function, helping patients with neuro-inflammatory or immune-related neuropsychiatric conditions^29,30^.

Greater understanding and use of endocannabinoids in medical practise is ever-increasing, adapting their application to many disease conditions, and to many medical fields. These disorders range from pain management in patients with cancer and chronic inflammatory disease, to intractable epilepsy in children, schizophrenia, and mental disease in psychiatry. Although initially utilized for analgesia and pain relief over a century ago, the *cannabis* extract-based medications, quickly fell out of favour due to their psychotropic side-effects which largely outweighed any benefits perceived at the time. Despite these views, a recent rekindled appeal for endocannabinoid usage has resulted in a resurgence of interest and usage in therapeutics as the consequence of a better grasp of their mode of action, receptor function, mode of delivery, identification of analogs without psychotropic effects, and public pressure prompted by the opioid pandemic. In summary, we believe this is the first report of targeted endocannabinoid-based NPs used as a drug delivery system with inbuilt endocannabinoid activity at the site of localization. This offers numerous advantages and the promise of a therapeutic in the treatment of inflammatory disease, chronic pain management, fibrosis, and neurological conditions including Alzheimer’s.

### Ethics

Approval was obtained from the Sydney Northern Area Health Animal (RNSH) Ethics Committee (Ethics Approval Number: RESP 15/15); and the Western Sydney Local Health District (WSLHD) Animal Ethics committee (Ethics Approval Number: 5105.08.12). The study was conducted in accordance with ARRIVE guidelines (https://arriveguidelines.org). Experiments performed at WSLHD Housing Facility and RNSH animal facility were in accordance with their animal ethics guidelines and regulations.

## Supplementary Information

Supplementary Information 1.

Supplementary Information 2.

Supplementary Information 3.

## Data Availability

The data discussed in this publication has been deposited in NCBI's Gene Expression Omnibus and are accessible through GEO Series accession number GSE206164 (https://www.ncbi.nlm.nih.gov/geo/query/acc.cgi?acc=GSE206164).

## Abbreviations

1-D: One-dimensional
2-D: Two-dimensional
2-AG: 2-Arachidonoylglycerol
AEA: Arachidonoyl ethanolamine (anandamide)
AECA: Arachidonyl-2-chloroethylamide
AGRF: Australian Genome Research Facility
AIA: Adjuvant induced arthritis
AP-1: Activator protein 1
BSA: Bovine serum albumin
CB_1_: Cannabinoid receptor 1
CB_2_: Cannabinoid receptor 2
CCL2/MCP-1: Monocyte chemoattractant protein-1
CNS: Central nervous system
COX-2: Cyclooxygenase-2
CP450: Cytochromes P450 monooxygenases
CPP: Critical packing parameter
Cryo-TEM: Cryogenic transmission electron microscopy
CXCR: C-X-C motif chemokine receptor
CXCL: C-X-C motif chemokine ligand
DAG: Diacylglycerol
DAGL: Diacylglycerol lipase
DCM: Dichloromethane
DE: Differentially expressed
DIC: Differential interference contrast
DiD: 1,1ʹ-Dioctadecyl-3,3,3ʹ,3ʹ-tetramethylindodicarbocyanine,4-chlorobenzenesulfonate salt
DLS: Dynamic light scattering
DSC: Differential scanning calorimetry
ERK1/2: Extracellular signal-regulated kinase-1/2
FAAH: Fatty acid amide hydrolase
FACs: Fluorescence activated cell sorting
FCS: Fetal calf serum
FLS: Fibroblast like synoviocytes
FT-NIR: Fourier transform near infrared spectroscopy
GM-CSF: Granulocyte-macrophage colony-stimulating factor
HAP-1: Synovium targeting peptide
HPLC: High performance liquid chromatography
HPLC/MS/MS: High performance liquid chromatography tandem mass spectrometry
IFN-ɣ: Interferon gamma
IL: Interleukin
IPA: Ingenuity pathway analysis
i.v.i: Intravenous injection
LEA: Linoleoylethanolamide
LN2: Liquid nitrogen
LXR: Liver X receptor
MMP: Metalloproteinase
MS: Mass spectroscopy
MTB: Mycobacterium tuberculosis
MRGPRX3: MAS related GPR family member X3
MYBL2: MYB proto-oncogene like 2
NAAA: *N-*Acylethanolamine-hydrolyzing acid amidase
NAEs: *N*-Acylethanolamines
NAPE: *N-*Arachidonoyl-phosphatidyl-ethanolamine
NAPE-PLD: *N*-Acylethanolamine-phosphatidyl ethanol amine
NF-κB: Nuclear factor kappa-light-chain-enhancer of activated B cells
NIR: Near-infrared spectroscopy
NOS2: Nitric oxide synthase 2
NP: Nanoparticle
NPnon-targeted: Non-targeted nanoparticle
NP_HAP_-1: HAP-1 conjugated nanoparticle
NP_sHAP-1_: SHAP-1 conjugated nanoparticle
OA: Osteoarthritis
OEA: Oleoylethanolamide
PBS: Phosphate buffered saline
PCA: Principal component analysis
PCR: Polymerase chain reaction
PE: Phosphatidylethanolamine
PEA: Palmitoylethanolamide
PEG: Polyethylene glycol
PEG2000: Polyethylene glycol-2000
PPAR: Peroxisome proliferator-activated receptors
RA: Rheumatoid arthritis
RES: Reticulo-endothelial system
RELA: NF-κβ p65 subunit
RELB: NFκβ heterodimeric p50/p52 subunit
RGD-PEG: RGD peptide-polyethylene glycol
RNSH: Royal north shore hospital
ROI: Region of interest
RP-HPLC: Reverse phase high performance liquid chromatography
RPP-HPLC: Reverse phase preparative high performance liquid chromatography
RT: Room temperature
RT-PCR: Reverse transcription polymerase chain reaction
RXR: Retinoid X receptor
SA-PE: Streptavidin, R-phycoerythrin conjugate
scFv: Single-chain variable fragment
SERPINB2: Serpin family B member 2
sHAP-1: Scrambled-synovium targeting peptide
STAT: Signal transducers and activators of transcription
TFA: Trifluoroacetic acid
TFIP2: Tissue factor pathway inhibitor 2
TNF-α: Tumor necrosis factor alpha
TLR: Toll like receptor
TRP: Transient receptor potential
TRPA1: Transient receptor potential cation channel subfamily A member 1
TRPV1: Transient receptor potential vanilloid 1
