# Supplementary material for: Design and function of targeted endocannabinoid nanoparticles
Source: Sci Rep. 2022 Oct 14;12:17260. doi: 10.1038/s41598-022-21715-1 (PMC9568518; doi:10.1038/s41598-022-21715-1)

**SUPPLEMENTARY FIGURE CAPTIONS**

**Figure 1**

MS analysis of (A) Ole-PEG2000-Succ (m.wt ~2362) (B) Ole-PEG2000-HAP-1 (m.wt ~3682) and **(C)** Ole-PEG2000-sHAP-1 (m.wt ~3682) confirmed the correct weight of the conjugated deprotected peptides.

**Figure 2**

(A) Dynamic light scattering of NP dispersions using a zetasizer is sized at 170 nm.

(B) Cryo-TEM image of NP dispersions prepared from 40% w/w OEA, 60% w/w LEA stabilized with 15% PEG2000-OH showing the production of sponge-like NPs – spongosomes.

(C) 1D SAXS patterns of NP dispersions with and without HAP-1 targeting. Assessment of the NP structure by SAXS supports the formation of sponge-like NP at 40% (w/w) OEA, 60% (w/w) LEA stabilized with 15% PEG2000-OH. Both dispersions showed colloidal particles with less ordered internal nanostructures, in contrast with the bulk lyotropic phase behavior described in Fig. 1D. The lack of highly ordered internal nanostructure is likely due to the addition of PEG lipids at 15wt %. Less PEG-lipid led to NPs with less stability over time. As shown in Fig 1D, the lyotropic mesophases were equilibrated for 48 hours and analysed with a laboratory SAXS instrument equipped with a Peltier temperature control chamber at 25°C (ii) and 37°C (iii). Lyotropic mesophases of 60% LEA at various temperatures, showing cubic mesophase of a Pn3m space group is shown in (iv). In Figure 1D (i) all the amphiphilic mixes examined showed a relatively sharp peak in addition to a broad peak. 100% LEA sharp peak at ~0.132 Å^-1^ is indicative of an ordered lamellar mesophase. As the percentage of OEA is increased, there is a shift in sharp peak to lower q values indicating a slight increase in the lattice parameter. Representative lyotropic mesophase of the hydrated mixed samples at 70wt% water (excess water) at 25°C are shown in (ii). The scattering pattern of various amphiphiles mixes ranging from 100% LEA to 50% LEA showed the formation of varying ordered nanostructures. The shift to lower q value from those observed in the neat amphiphile is indicative of a swollen L_2_ mesophase with a weakly ordered nanostructure. As LEA ratio is increased, the molten mesophase changed slightly and a sharp peak appeared at 0.147 Å^-1^_._  At 70% LEA a mixed cubic mesophase, along with a crystalline lamellar peak at q=0.143 Å^-1^ was observed. At 60% LEA, the cubic mesophase became more dominant however transformed to a mixed cubic and lamellar mesophase at 50% LEA.

The lyotropic mesophases of the hydrated mixes was also assessed at 37°C (iii). Raising the temperature of the above hydrated samples to physiological temperature resulted in more molten L_2_ mesophase for mixes containing 90-70% LEA. However, at 60% LEA, a mono cubic mesophase with the Pn3m symmetry was developed. At 50% LEA, there is a shift from sole cubic mesophases (as seen in 60% LEA), with both cubic mesophase mixed with a lamellar liquid crystalline mesophase observed. From this study, we found that among all the various ratios investigated here, 60% LEA mixed amphiphile showed a sole cubic mesophase of Pn3m symmetry, which was stable up to physiological temperature. Figure 1D (iv) shows the shift in lattice parameter of 60% LEA mix. The lattice parameter of this mesophase decreased as the temperature increased, by shifting the scattering peaks towards higher q values.

**Figure 3**

(A) WST-1 results determining NP *in vitro* cytotoxicity. LC50 value is frequently used as a general indicator of a substance's acute toxicity. At 30 µg/ml, approximately 75% of cells were still viable and chosen as the standard concentration of NP to be used in all *in vitro* experiments.

(B) Confocal fluorescent microscopy images of HAP-1-binding to HIG-82 cells. Cells were incubated with media, HAP-1-biotin, or sHAP-1-biotin at 37˚C followed by streptavidin-FITC. Actin was labelled with TRITC-phalloidin shown as red (a), HAP-1-biotin labelled with streptavidin-FITC (green, b), and nuclei labelled with DAPI (blue, c). Panel (d) shows HIG cell with actin and cytoplasmic HAP-1. Scale bars represent 100 µm.

(C) Flow cytometry histograms illustrating cell fluorescence as an indicator of NP dye stability following incubation with NP_non-targeted_ and NP_HAP-1_ at 4°C and 37°C. Autofluorescence (red), incubation at 4°C (green), and incubation at 37°C (blue). The absence of cell fluorescence at 4°C indicates successful retention of dye within the NP. Experiments were repeated three times. The shown histogram is a representative example**.**

**Figure 4**

Pharmacokinetic profiles for (A) NP_non-targeted_ and (B) NP_HAP-1_ content vs time (up to 6 hours) fitted with a one-phase exponential decay curve. The initial concentration of total OEA in plasma was 214 ± 84.3 µmol/g and LEA 143.5 ± 47.2 µmol/g following injection as extrapolated from the exponential decay and decreased bi-exponentially after the 6 hour sample. The PK profiles of total NP in the plasma from 0 – 6 hours were fit using a one-phase decay non-linear regression curve. The terminal half-life for NP_non-targeted_ was 0.16 hours (LEA), 0.20 hours (OEA). (B) The half-life for NP_HAP-1_ 0.31 hours (LEA) 0.48 hours (OEA). Tissue:plasma ratio of OEA and LEA in (C) NP_non-targeted_ and; (D) NP_HAP-1_ treated arthritic rats showing accumulation of NP to the inflamed paw at 6 hours (mean, n = 5).

**Figure 5**

(A) PCA plots and (B) sample-sample distance heat maps of RNA-seq data from RA-FLS cells; RA_UT, RA_NP, RA_TNF, RA_TNF_NP. RA-FLS samples clustering met assurance quality. The number of clusters, along with the percentage variance between the clusters was reported and confirmed that the samples clustered within their respective treatment groups, as expected.

**Figure 6**

Heat map of the top DE genes based on comparison of RNA-seq data between TNF-α and NP treated cells (RA-TNF/NP), and TNF-α treated (RA-TNF) RA-FLS cells. The normalized RNA-Seq data is in log2 scale, where red is highly expressed genes and blue is low expression. To be included in the heat map, genes were required to have at least 1000 counts (reads), totalled over all samples, where the standard deviation of log2 expression differences had to exceed two. The heatmap highlights an immunological shift from highly pro-inflammatory in an acute inflammatory environment, mediated by NP.

**Figure 7**

Network pathways of DE expressed genes in (A) Macrophages, Fibroblasts and Endothelial Cells in Rheumatoid Arthritis (B) Hepatic Fibrosis/Hepatic Stellate Cell Activation (C) LXR/RXR Activation. Activation states are taken from DE genes obtained from RNA-seq data of RA-TNF/NF treated cells compared to RA-TNF treated cells. Green represents low expression; red represents high expression.


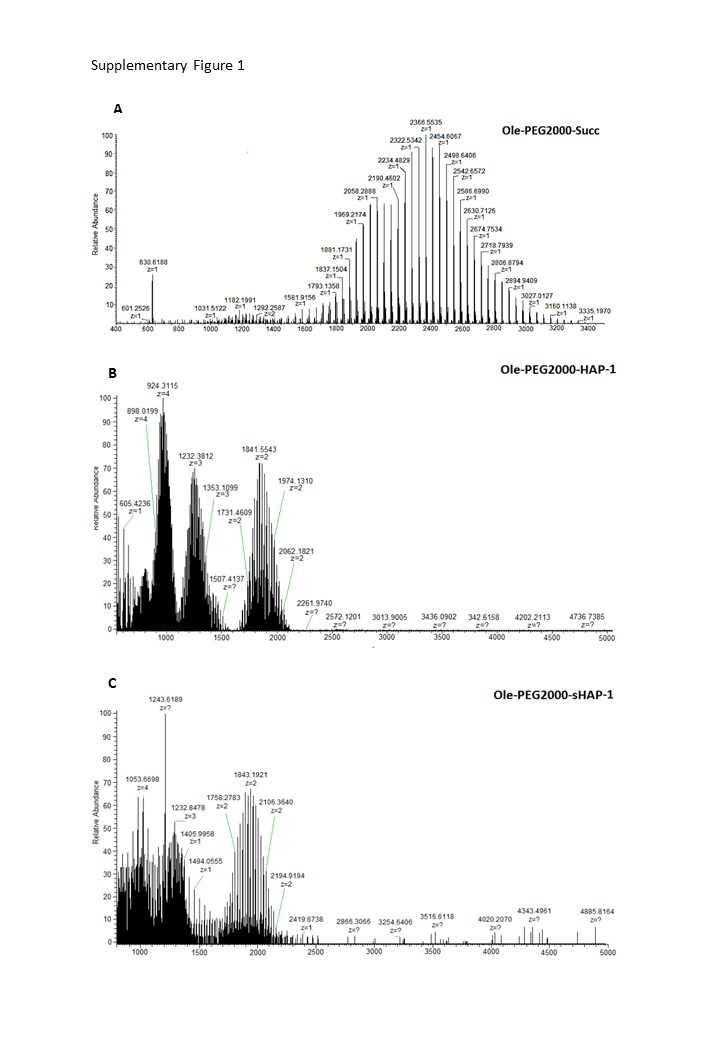


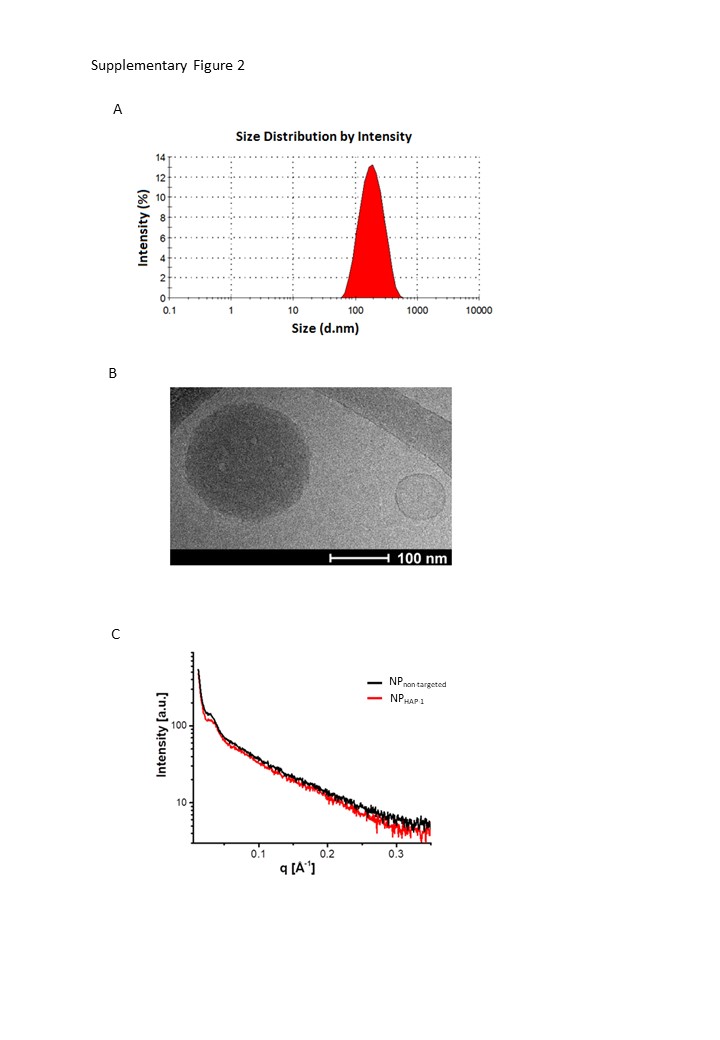


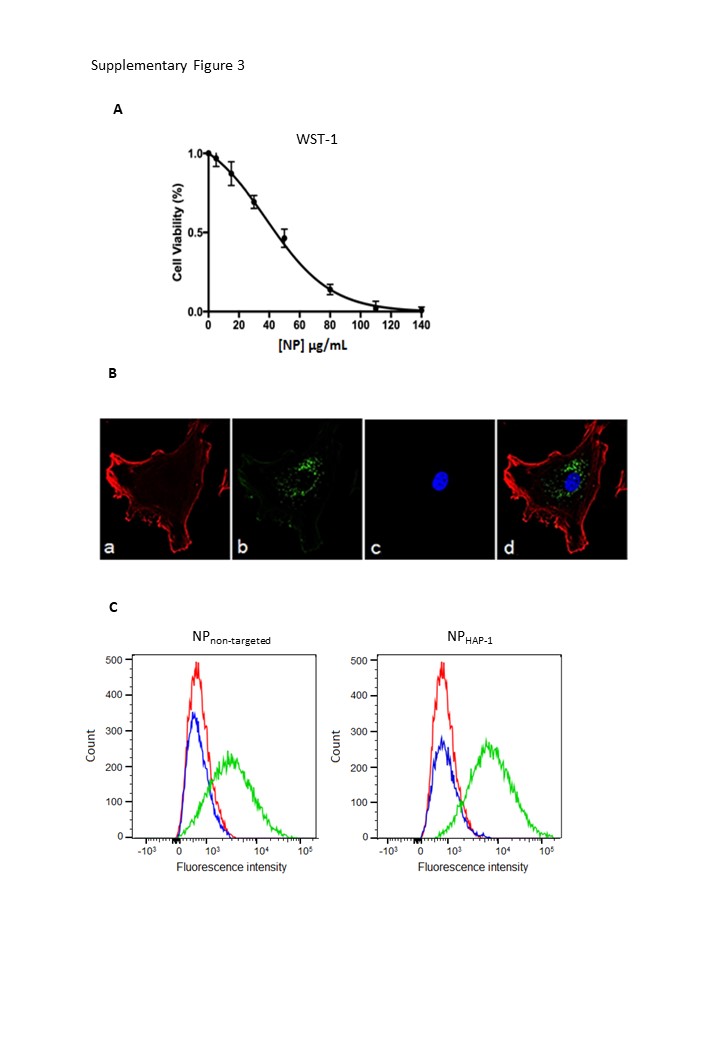


**
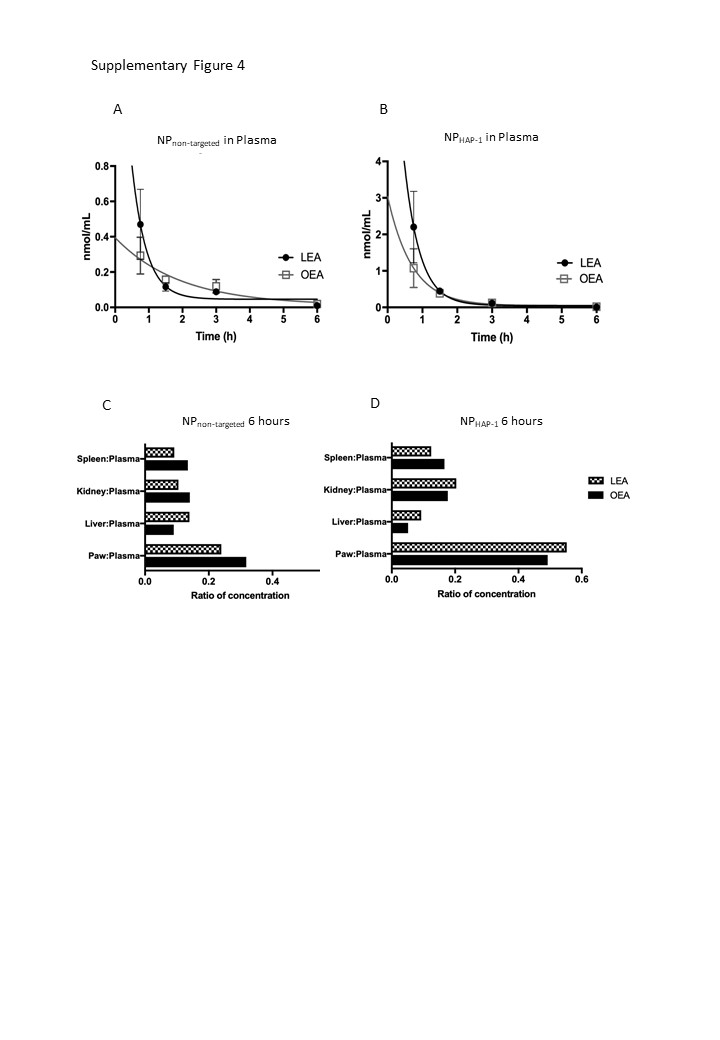
**


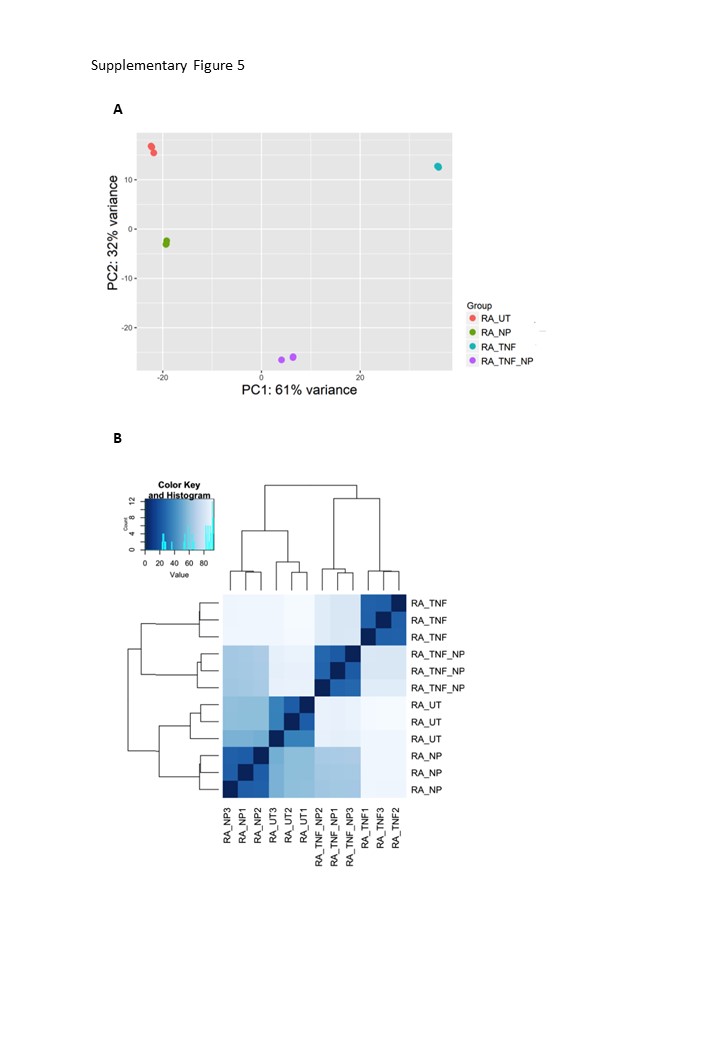


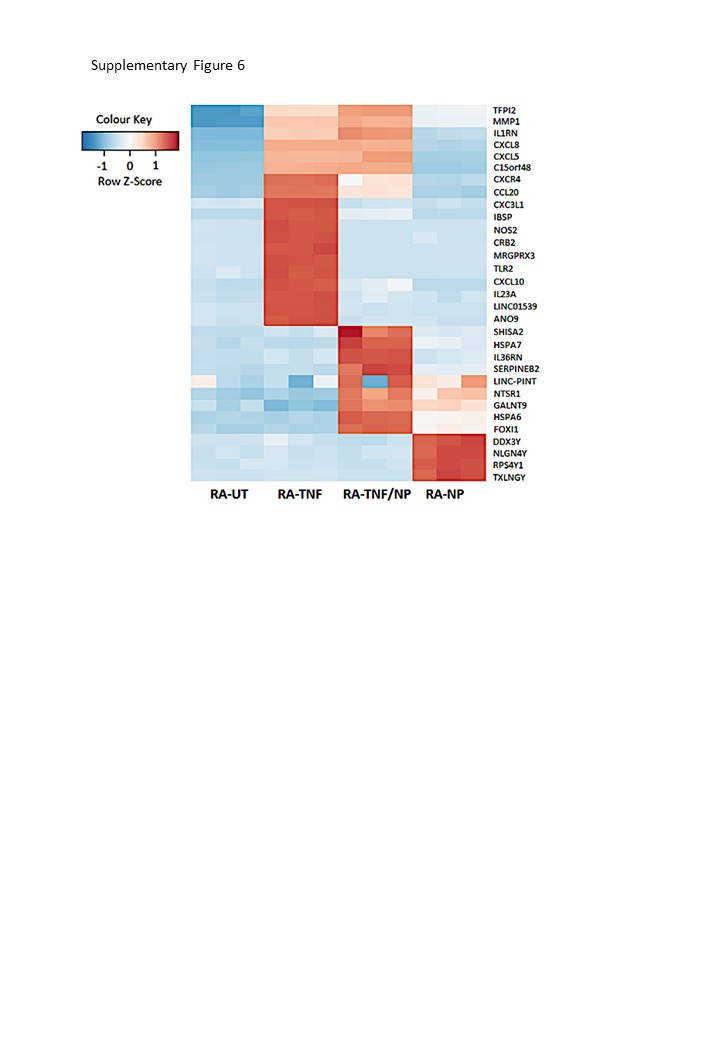


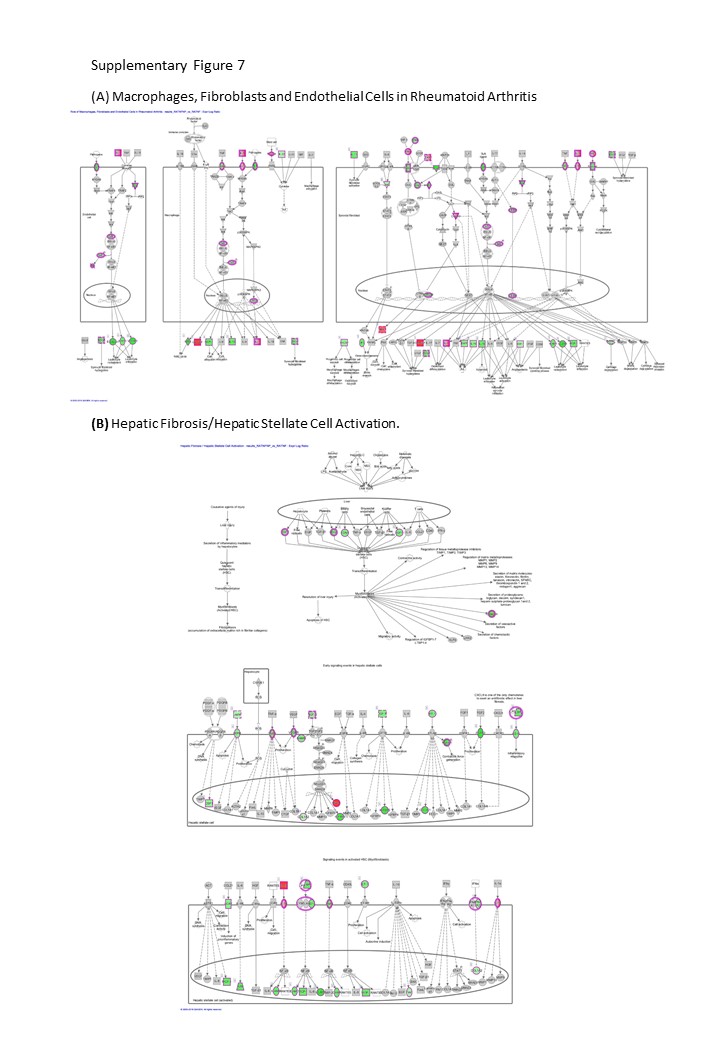


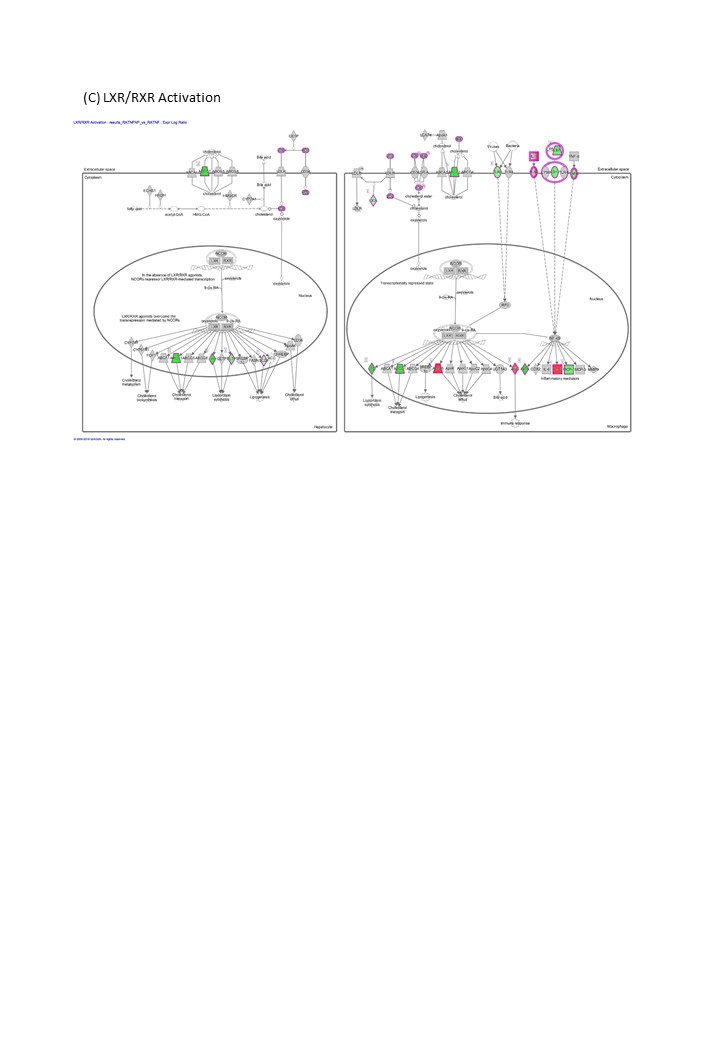

Supplement: Supplementary file 1 — Supplementary Information 1. [file 41598_2022_21715_MOESM1_ESM.docx]
