# Supplementary material for: Design and function of targeted endocannabinoid nanoparticles
Source: Sci Rep. 2022 Oct 14;12:17260. doi: 10.1038/s41598-022-21715-1 (PMC9568518; doi:10.1038/s41598-022-21715-1)
Supplement: Supplementary file 2 — Supplementary Information 2. [file 41598_2022_21715_MOESM2_ESM.docx]

SUPPLEMENTARY MATERIALS AND METHODS

**Materials**

Targeting and biotinylated peptides were custom synthesised by Auspep (Melbourne, Australia). Purchased peptides were shown to be of high purity (>80%) by Reverse Phase High Performance Liquid Chromatography (RP-HPLC) and characterised by a Mass Spectrometer (MS) equipped with a matrix assisted laser desorption/ionisation (MALDI) source (Applied Biosystems Inc., Foster City, CA). Organic solvents and other reagents were purchased from Sigma-Aldrich (Sydney, Australia) and were of analytic or spectroscopic grade and used as received. A Milli-Q Plus Ultrapure water system (Millipore, Australia) was used to filter deionized tap water to obtain high purity water.

**Methods**

Monoethanolamide lipid synthesis. The desired fatty acid (oleic acid: 20 mmol, 5.65 g) was dissolved in dichloromethane (DCM) in a round-bottom flask and stirred vigorously on ice. Oxalyl chloride (40 mmol, 5 g) was then added to the resulting solution and the reaction stirred for a further 10 minutes on ice. The flask was then sealed, and the reaction mixture was stirred for 2 hours at room temperature (RT). The solvent and oxalyl chloride were removed under vacuum using a rotary evaporator (Rotavapor R-210; Buchi Instruments, Germany). The resulting fatty acyl chloride was dissolved in DCM and slowly added drop wise into an ethanolamine solution (40 mmol, 2.44 g) in DCM. The reaction was maintained in an ice bath with rapid stirring. After 10 minutes, the reaction was returned to RT and stirred for 2 hours. The resulting product was filtered using Whatman 542 filter paper. The filtered solution was then sequentially rinsed with 4% citric acid, 4% sodium bicarbonate solution, and Milli-Q water. DCM was then evaporated under vacuum, leaving a white powder for OEA. Similar procedure was used for the synthesis of LEA to yield an oily residue for the LEA. Both NAEs synthesis had similar yields of greater than 95%.

Synthesis of **ole-PEG2000-succ.** The excess solvent and oxalyl chloride were removed under vacuum using a rotary evaporator. 1-2 ml of DCM was added to the residue and removed via rotary evaporator. This process was repeated three times to eliminate any excess oxalyl chloride. The oleoyl chloride (2.1 mmol, 0.65 g) was solubilised in DCM and added to a PEG2000 (1.4 mmol, 2.7g) solution dissolved in 20 mL anhydrous DCM. The pH of the reaction was adjusted using triethylamine (TEA) to achieve a pH 8-9. The mixture was then stirred for 2 hours at room temperature and the solvent evaporated. Prepared samples were stored at 4˚C prior to purification by reverse phase preparative HPLC (RPP-HPLC). Ole-PEG2000-OH had a total yield 78.5%. For succinylation of Ole-PEG2000-OH, succinic anhydride (344 mmol, 0.344 g) dissolved in 5 mL acetonitrile was added to a PEG2000-Oleate (86 mmol, 1.72g) solution in 20 mL acetonitrile. TEA was added to adjust the pH to 9 and the reaction stirred at room temperature overnight. The solvent was evaporated under reduced pressure and purified on the RPP-HPLC. Ole-PEG2000-Succ had a total yield 76%.

**Ole-PEG2000-Succ linkage to targeting peptides HAP-1 and sHAP-1.** Ole-PEG2000-Succ (0.07465 mmol, 176.96 mg) was dissolved in 10 mL acetonitrile (ACCN). **2-(1H-Benzotriazole-1-yl)-1,1,3,3-tetramethylaminium tetrafluoroborate (TBTU;** 0.1497 mmols, 48.1 mg) dissolved in ACCN was added to an Ole-PEG2000-Succ solution and stirred for 30 minutes at room temperature. For activation, one mole equivalent of N, *N-*diisopropylethylamine (DIEA; 0.07485 mmol, 48.1 mg) was added to the Ole-PEG2000-Succ solution and stirred for 2hours at room temperature. HPLC was then followed to monitor the reaction process. Following activation, 100 mg of protected peptide dissolved in DCM was added in a 1:1 ratio to the solution and stirred for 2 hours at room temperature. The pH was checked and adjusted to 8 by addition of TEA. All the solvents were removed under vacuum using a rotary evaporator. The protecting groups of the conjugated peptide were then cleaved off using a mixture of trifluoroacetic acid (TFA), water and triisopropylsilane in the ratio of 95:2.5:2.5, respectively. The solution was stirred for 2 hours at room temperature and the solvents removed via a rotary evaporator. Ether was added and re-evaporated three times to remove any remaining TFA. The conjugated HAP-1 and sHAP-1 peptide-PEG oleates were then purified respectively using the Reveleris flash chromatography and a C18 wide pore column and using a linear gradient solvent system: solvent A: 90% v/v water-10% v/v ethanol, and solvent B: 100% v/v ethanol. The pure fractions were pooled, evaporated to dryness, and lyophilised by dissolving the residue in tertiary butyl alcohol (TBA) and freeze drying overnight. The final samples were tested using Thermo Fisher scientific Exactive plus MAX LC/MS and an ESI probe. Final sample yields were 32.5% for Ole-PEG2K-HAP-1 and 41.1% for Ole-PEG2K-sHAP-1.

**Peptide purity assessment.** The purity of the peptide was assessed by analytical HPLC using a Vydac C18 reverse-phase column on an Agilent 1100 series HPLC system. Liquid chromatography–mass spectrometry (LC/MS) were used to assess molecular weight of final synthesised and conjugated peptides. For LC/MS, samples were dissolved in pure LC-MS grade methanol and analysed using a Thermo Scientific Quadrupole Orbitrap (Q-exactive plus) kindly provided on loan by Thermo Scientific Australia (Sydney, Australia). Samples were run on positive ion scan with MS range 200-6000 m/z on high resolution. MS of synthesised samples is shown in Supplementary Fig. 1. Nuclear magnetic resonance spectroscopy (NMR) scans were then used to assess the final molecular structure of the synthesised peptides. For NMR, Samples were dissolved in chloroform-d (CDCl_3_) to a final concentration of 15 mg/ml and measured on a 400 T Brucker NMR at 25 °C and referenced internally to the solvent. Analysis of monoethanolamide lipids, for signal assignment and purity assessment, were carried out using MnNova software.

***In vitro* studies**

**WST-assay.** Human (h)-FLS cells were seeded at 1 x 10^4^ into 96-well plates (Corning, CoStar, Sigma-Aldrich, Sydney, NSW) and cultured overnight (80% confluence) prior to treatment. After seeding, the medium was replaced, and various concentrations of NP were added to the microtiter wells and incubated at 37°C for 24 hours. Following incubation, 10 µL of WST-1 was then added and incubated at 37°C for 4 hours and the absorbance measured using an ELISA plate reader. The quantity of formazan dye was determined by the absorbance at 450 nm with a reference wavelength at 630 nm is directly proportional to the percentage of viable cells. Control values (wells without NP stimuli or PBS vehicle control) were set at 100% viable. The relative cell viability (%) related to control wells was calculated by [A]test/[A]control × 100. Where [A]test is the absorbance of the test sample and [A]control is the absorbance of control sample. To make the technicality correction, a background control (blank) was provided for every treatment group to preclude the potential interference of the NP and medium with the spectrophotometric measurement. Samples were run in triplicates and repeated n = 3, and respective TC-50 (particle concentration inducing 50% cell mortality) concentrations were calculated by regression analysis using GraphPad Prism software.

**Quantification of NP-cell complexes by flow cytometry.** Fluorescence from cells was measured using BD FACS Cantroll analytic flow cytometer (BD Bioscience, San Jose, CA) and acquired with FACSDiVa v.6 software. DiD-NP were excited at 644 nm, and fluorescence emission detected at 665 nm using a 20-nm band-pass filter. A minimum 500,000 cells were counted per sample. Selective exclusion of cellular fragments and debris from analysis was achieved by the subjective collection gating from distribution in the side-scatter versus forward scatter dot plot. To determine the extent of DiD association with cells, a histogram was drawn with the x-axis set for cell fluorescence (detection of DiD) and the y-axis set to cell count. Settings for the flow cytometry machine was kept constant between the incubation times for both cell lines to ensure that mean intensity acquired by the gated peaks were constant. Collected data was analysed using FlowJo.

***In vivo* studies**

**Animals.** Female Wistar rats (240 – 250 g, 8 to 9 weeks old) were purchased from Animal Resources Centre (Perth, WA, Australia) and were housed in Kolling Institute’s Kearn’s Animal Facility located within Royal North Shore Hospital, Sydney. Rats were housed three per cage and provided with standard lab chow and water *ad libitum.* Rats were left to acclimatise for 2 weeks prior to the induction of any experiment. Experiments performed were in accordance with Sydney Northern Area Health Animal Ethics guidelines (ethics approval number: RESP 15/15). Similar conditions were present in the Westmead Housing Facility and experiments performed in accordance with Western Sydney Local Health District Animal Ethics guidelines (ethics approval number: 5105.08.12).

**Localisation of NPs.** To assess NP localisation *in-vivo*, fluorescently labelled NP_non-targeted_, NP_HAP-1_ and NP_sHAP-1_ were intravenously injected (i.v.i) into the tail vein of both normal and arthritic rats and localisation tracked using a NIR imager. Rats were divided into three groups of five and administered with either NP_non-targeted_, NP_HAP-1_ or NP_sHAP-1_, intravenously. Rats were anaesthetized under isoflurane/oxygen (2% v/v isoflurane in 1 litre/min O_2_) and NP (12 mg/kg) administered once, i.v.i. The animals remained anaesthetised and placed prone on a gamma camera (double head; Siemens Medical Systems, IL, USA) equipped with a low-energy high-resolution collimator. NP *in-vivo* localisation was captured 24 hours for 10 minutes using Fourier Transform Near Infrared (FT-NIR) spectrometer (Bruker, Victoria, Australia). The images were acquired using the following parameters, matrix: 256 x 256 and zoom: 2.29. For optimum DiD intensity, emission was taken at 700 nm with a corresponding excitation of 650 nm. Program ‘Image J’ (Version 1.51) was used on the region of interest (ROI) on the chosen arthritic joint of each rat for each time point. X-rays were used to confirm the anatomical position of the region for a single case. The values reported were obtained by first averaging the signal value determined in the joints then by subtracting the background value from the signal obtained at each time-point for each joint.

**Pharmacokinetics and NP distribution in rats. T**issue samples were weighed and HPLC grade methanol (MeOH) added to each tube to make a 100 mg/mL solution. For plasma, 3 mL of cold acetonitrile was added to precipitate proteins. Internal standard, deuterium labelled anandamide (d4-AEA), was added to tissue samples (100 µL of 1 µM) and plasma samples (10 µM of 1 µM) and incubated on ice for 2 hours (plasma) to 12 hours (tissues). Tissues were homogenised on ice for approximately 2 minutes using a tissue tearer, except for the paw joints which were bead beaten (glass beads 2.5mm) for 2 minutes at 35,000 oscillations/minute. The samples were then centrifuged at 19,000 x *g* for 20 minutes at 24°C. Tissues were extracted in duplicates of 500 µL (spleen, liver) or 1 mL (kidney, paw) of each supernatant. HPLC-grade water was then added to make the final supernatant (tissue and plasma) solution 25% organic. Samples were extracted through The Extrahera (Biotage, Uppsala, Sweden), an automated extraction robot through 500 mg C18 solid phase extraction columns. The columns were conditioned with 5 mL of HPLC MeOH (170 sec of 0.5 bar) and 3 mL of HPLC grade water (80 sec of 0.5 bar). 25% of organic supernatant solution was then loaded into the corresponding column. Wash steps of 1.5 mL of HPLC water, 40% MeOH 65% MeOH, and 85% MeOH (90 sec each at 0.5 bar) were then added in succession. 1.5 mL of 100% HPLC grade MeOH (105 sec at 0.1 bar, with plate dry) was then added to elute the ethanolamides which was collected in amber autosampler vials. Vials were then put straight into the autosampler (24⁰C).

**Quantification of inflammatory cytokines in circulating rat plasma.**  Collected blood was centrifuge for 10 minutes at 1,000 x *g* and the plasma separated, snap frozen in liquid nitrogen and stored at -80°C prior to analysis. Plasma cytokines were quantified using an LEGENDplex™ Rat Th Cytokine Panel (13-plex), immune-bead based assay, detecting IL-10, IFN-γ, CXCL1/KC, CCL2/MCP, TNFα, GM-CSF, IL-18, IL-12p70, IL-1β, IL-17A, IL-33, IL-1α and IL-6 (Biolegend, San Diego, CA, USA). Analysis was performed using BD FACS Cantroll analytic flow cytometer (BD Bioscience, San Jose, CA, USA) according to the manufacturer’s instructions for analysis in plasma samples. Data analysis was performed using LEGENDplex™ software (Biolegend, Australian Biosearch, Karrinyup, WA, Australia), version 7.1 and presented as concentrations (pg/mL).

# Endocannabinoid NP localisation. NP *in vitro* cell cytotoxicity was assessed by colorimetric assay WST-1 as shown in Supplementary Fig 3A. The LC50 was 40 µg/mL after a 24 hour incubation period and NPs were subsequently used at 30 μg/mL concentration. To determine *in vitro* homing peptide HAP-1 binding and internalisation into FLS cells, biotin-conjugated HAP-1 and controls were added to HIG-82 (rabbit), human (h) osteoarthritic and rheumatoid arthritic (OA and RA, respectively) derived FLS followed by avidin-FITC staining. Confocal images of HAP-1 binding to HIG-82 cells are shown in Supplementary Fig 3B. A similar staining pattern was noted with RA-FLS and OA-FLS cell types (Fig. 2B). To evaluate the FLS binding and uptake of NPs_,_ fluorescent labelled NP_non-targeted_ and NP_HAP-1_ were incubated with h-FLS cells at 37˚C for 1-3 hours and signal fluorescence assessed using flow cytometry. As shown in Fig. 2B (i, iv) there was no detectable free dye or leakage recorded for NP_non-targeted_ and NP_HAP-1_ following 1 hour incubation with HIG-82/h-FLS cells. Following 3 hour incubation (ii, v), NP_HAP-1_ was internalised more efficiently than NP_non-targeted_ and the cell fluorescence peak shifted to higher intensity, with the percentage of cells that took up detectable dye recorded as 69.1% for NP_HAP-1_ and 33.2% for NP_non-targeted_. Increasing NP contact to 18 hours (iii, vi) improved uptake of both NP_HAP-1_ and NP_non-targeted_ to 74.5% and 44.8%, respectively, when compared to 3 hour exposure. Cell fluorescence at 4˚C and 37˚C for 3 hours is shown in Supplementary Fig 3C. At 4˚C there was no fluorescence from cells incubated with either DiD labelled NP_non-targeted_ or DiD labelled NP_HAP-1_ suggesting that the encapsulated DiD dye was retained within the NP. Conjugation of HAP-1 to the NPs facilitated the preferential uptake of NP_HAP-1_ by h-FLS cells when compared to NP_non-targeted_ *in vitro* suggesting a receptor-mediated process.

Extending the *in vitro* studies, fluorescent-tagged NPs were injected into normal and arthritic rats and their localisation tracked using a NIR imager. NP accumulation was measured as fluorescence intensity and captured at 1, 3, 24 and 48 hours for 10 minutes (Fig. 3A). After 24 hours, NIR fluorescence signals remained constant. There was selective accumulation of the NP_HAP-1_ to joints not observed with either the NP_non-targeted_ or NP_sHAP-1_. In normal rats, no specific accumulation of fluorescence was seen for the NP_non-targeted_ rats. Similarly, normal rats administered NP_sHAP-1_ showed no detectable localisation to the joints (21.37 ± 4.83 signal units). In arthritic rats, localisation of both the NP_non-targeted_ and the NP_sHAP-1_ to the affected joints was increased by 62.1% (32.1 ± 8.24 signal units) and 71.2% (36.06 ± 5.57 signal units) respectively. Signal units for unaffected joints of the arthritic rats were comparable to the joints in normal rats. The restricted accumulation of NP_non-targeted_ and the NP_sHAP-1_ to only the affected arthritic joints could be attributed to passive targeting accredited to the leaky vasculature of the inflamed area. In contrast to the NP_non-targeted_ and NP_sHAP-1_ treated groups, normal rats injected with the targeted NP_HAP_ localised to joints (34.37 ± 2.08 signal units). Signal at these joints was 73.6% higher than that observed in NP_non-targeted_ (19.79 ± 6.04 signal units) normal rats. In arthritic rats, localisation of NP_HAP-1_ to the inflamed joints increased 58.2% (54.37 ± 13.95 signal units) in comparison to NP_HAP-1_ treated normal rats and 69.3% higher than NP_non-targeted_ arthritic rats. Following whole-animal imaging at 24 h, the rats were sacrificed and the major internal organs; spleen, liver, kidneys, heart, and lungs, harvested for NIR imaging (Figure 3B). Minimal fluorescence was observed in the heart, kidneys, spleen, and lungs. In contrast, strong signal was noted in liver of the rats suggesting greater clearance of the NP at these sites. While the organs fluorescence was comparable between the two NP groups, fluorescence of the liver from the NP_non-targeted_ group was significantly higher when compared to the liver of the NP_HAP_ injected group.

# Biodistribution of targeted and non-targeted NPs. In addition to measuring blood plasma levels of OEA and LEA from normal (NORM), untreated arthritic rats (ART CON), and arthritic treated rats, endogenous NAE levels and their entourage compounds, NAEA, 2-AG, PEA plasma were also measured. To assess the NPs pharmacological half-life in arthritic rats, NPs were administered via the tail vein and blood collected at 45 minutes, 1.5, 3, and 6 hours. The distribution of NP OEA and LEA lipids in solid organs was determined at 6 h post NP administration (Figure 4C, D), measured against baseline levels of control arthritic rats. For NP_non-targeted_ treated rats, the liver contained the highest relative concentration of OEA (1.72 ± 0.87 pmol/g) and LEA (9.38 ± 5.2 pmol/g) when compared to endogenous ART-CON levels. Bio-distribution of NP_non-targeted_ to the kidneys (OEA (2.69 ± 1.19 pmol/g) and LEA (0.98 ± 0.29 pmol/g)) and spleen (OEA (2.56 ± 1.02 pmol/g) and LEA (0.86 ± 0.29 pmol/g)) were minimal, with comparable concentrations to baseline ART-CON levels. While concentrations of NP_non-targeted_ were high in the liver, only small amounts of OEA (0.86 ± 0.09 pmol/g) and LEA (0.59 ± 0.10 pmol/g) were recorded in the liver for NP_HAP-1_ treated groups. These concentrations were much lower than that observed for NP_non-targeted_ and were in agreement with the lower fluorescence noted in the liver from NP_HAP-1_ treated rats imaged by NIR. Similarly, NP localisation to the kidneys (OEA (2.97 ± 1.30 pmol/g) and LEA (1.29 ± 0.57 pmol/g)) and spleen ((2.79 ± 1.58 pmol/g) and LEA (0.79 ± 0.219 pmol/g)) was minimal and comparable to that seen in NP_non-targeted_.

In the paw, high expression of OEA and LEA indicated localisation of NPs to the inflamed joint in both NP_non-targeted_ and NP_HAP-1_ injected rats (Fig. 4C,D). While both NP group constituents appeared to localise to the joints, only NP_HAP-1_ was significant for OEA (8.26 pmol/g of tissue, p < 0.05) and LEA (4.16 pmol/g of tissue, p < 0.05). By contrast, slightly higher plasma concentrations of OEA and LEA in the NP_non-targeted_ group coincided with reduced non-significant concentrations of OEA and LEA measure in the paw. Tissue: plasma ratio of OEA and LEA in NP_non-targeted_ and NP_HAP-1_ treated arthritic rats is illustrated in Fig. 4 (C) and (D), respectively. This data agrees with the localization results obtained by NIR, which indicated a significant uptake of NP_HAP-1_ in comparison to NP_non-targeted_ by the arthritic joints.

In addition, NP regulation of local joint endocannabinoids PEA, 2-AG and AEA was also assessed (Fig. 4E). Significant concentrations of OEA and LEA in NP_HAP-1_ treated group were correlated with significant increases in PEA (12.26 ± 10.27 pmol/g, p-value 0.0393). Elevated levels of PEA were also recorded for NP_non-targeted_ (9.13 ± 6.85 pmol/g, p-value 0.0409) treated rats, however, were not significant when compared to baseline levels. Similarly, levels of AEA were slightly increased in both NP_HAP-1_ (0.78 ± 0.65 pmol/g, p-value 0.1784) and NP_non-targeted_ (0.64 ± 0.48 pmol/g, p-value 0.0689) treated rats when compared to ART CON (0.07 ± 0.01 pmol/g), however these levels were not significant. In contrast to PEA, endogenous levels of 2-AG remained relatively unaffected in both NP_HAP-1_ (38.84 ± 10.86 pmol/g) and NP_non-targeted_ (41.92 ± 18.91 pmol/g) treated rats when compared to ART CON (41.68 ± 10.32 pmol/g). Collectively, the data suggests that NP’s endocannabinoid components LEA and OEA, regulate endogenous endocannabinoid levels.

**RNA-seq**

**Stimulation of human RA-FLS cells and RNA isolation.** RA-FLS were cultured in synoviocyte growth medium at 37°C and 5% CO_2_. Treated groups were run using three biological replicates. To study the effects of NP on inflammatory cytokine production, RA-FLS cells were stimulated with TNF-α alone (10 ng/ml, RA-TNF), or in the presence of endocannabinoid-NP (30 μg/mL, RA-TNF/NP) for 48 hours at 37°C. Untreated RA-FLS cells (RA-UT) and RA-FLS cells incubated with NP alone (30 μg/mL, RA-NP) were used as controls. Following incubation, cells were washed once with PBS and RNA isolated. Total RNA was isolated from cultured RA-FLS cells using Isolate II RNA Mini Kit (Bioline, Alexandria, Australia) according to the manufacturer’s instructions. To remove any genomic DNA contamination, RNA was treated with RNAse-free DNAse I (Sigma-Aldrich Pty, Sydney, Australia). RNA purity and concentration was assessed using Agilent 2100 Bioanalyser®(Agilent Technologies, Santa Clara, CA, USA).

|  |  |
| --- | --- |
|  |  |

**RNA-seq library construction and sequencing.** Stranded RNA libraries were prepared from 350 ng RNA using the Illumina® TruSeq Stranded mRNA sample prep kit. The libraries were sequenced by the Australian Genome Research Facility on an Illumina HiSeq 2500 to generate 50 base-pair (bp) single-end reads. Raw sequencing reads were assessed for quality using FastQC (version 0.11.3) (Babraham Bioinformatics), ensuring that Phred scores were over 30. Per base quantity scores were confirmed to be high using FastQC, and therefore no adaptor trimmings were performed. STAR (version 2.5.2a) was used to align the reads to Release 19 of the human reference genome (GRCh37/hg19), with the GENCODE (Release 28) annotation (<http://www.gencodegenes.org/)> provided, using default parameters. Resulting SAM files were sorted by position using SAMtools (version 1.6). Quality assessment, mapping and raw read counts were conducted on the high-performance computing cluster (Artemis), provided by the Sydney Informatics Hub, University of Sydney.

**Differential gene expression analysis.** Raw gene-level read counts were obtained using HTSeq (version 0.9.1) in “union” mode to exclude multi-mapping or ambiguously aligned reads. Raw counts were analysed using the DESeq2 (release 3.6) statistical package in R Studio (version 1.1.383). Data were transformed using the rlogTransformation function in DESeq2 to obtain principal component analysis (PCA) plots and sample-sample distance heatmaps. These plots were used to confirm that samples clustered within their respective treatment groups as expected. DESeq2 performs differential expression analysis on raw count data using a negative binomial distribution model. The model includes implementation of normalisation to correct for library size and independent filtering to exclude genes with low read counts. We defined genes as differentially expressed if FDR <0.05 (False discovery rate for multiple testing) and if log fold change > 2. Gene ontologies, pathways and regulatory networks that were enriched/overrepresented in significantly differentially expressed genes were identified using the Ingenuity Pathway Analysis (IPA, QIAGEN Redwood City, www.qiagen.com/ingenuity) software.

**Reverse transcription-polymerase chain reactions (RT-PCR).** RT-PCR was used to evaluate anti-inflammatory gene regulation in RA-FLS cells incubated with NP and confirm RNA-seq data. Total RNA from each sample was reversed transcribed into complementary DNA (cDNA) using SensiFast cDNA synthesis kit (Bioline, Alexandria, AUS) according to the manufacturer’s instructions. Briefly, a 20 µL reaction containing up to 1 µg mRNA, 5x TransAmp Buffer (4 µL), Reverse transcriptase (1 µL) were prepared for each sample. No reverse transcriptase controls were prepared from the untreated cells RNA and no template controls were prepared in water in place of total RNA to indicate potential genomic DNA contamination. RNA was reversed transcribed into cDNA using Thermocycler (Hybaid Omn-E, Hybaid Ltd) following, primer annealing at 25˚C for 10 mins, reverse transcription at 42 ˚C for 15 minutes, inactivation at 85˚C for 5 minutes and finally held at 4 ˚C.

Following reverse transcription, cDNA was subject to quantitative-Polymerase chain reaction (q-PCR) carried out on CFX96 RT-PCR detection system (Bio-Rad, Gladesville, NSW, Australia). PCR amplification was performed using specific primers IL-6 (F: GTGGG CGCCCCAGGCACCA, R: CTCCTTAATGTCACGCACCATTTC), IL-8 (F: ACTGAGAGTGATTGAGAGTGGAC, R: AACCCTCTGCACCCAGTTTTC), NFKB (F: GGTGCGGCTCATGTTTACAG, R: GATGGCGTCTGATACCACGG) . Primer sets were design using the Primer3web software and supplied by Integrated DNA-technologies (IDT, Baulkham Hills, NSW, AUS). PCR amplification was performed with SensiFast SYBR Green No-ROX kit (Bioline, Alexandria, Australia) according to established protocols. Briefly, 10 ul of 2x SensiFAST SYBR No-ROX mix, 0.8 μL 5′ sense primer and 0.8 μL 3′ antisense primer (10 μM, final concentration 400 nM), RNAse free water and template mixed in a 20 μL reaction were prepared. Samples were heated at 95˚C for 3 minutes and cycled for a maximum of 39 times. Each cycle included denaturation at 94°C for 30 seconds, annealing at 60 - 65°C for 10 seconds and extension at 72°C for 2 minutes. The housekeeping gene, GAPDH, was used as an internal control to normalise the amounts of mRNA in each sample. Quantification of mRNA levels was performed using the ΔΔCt method. The value of each control sample was set at one and used to calculate the fold change of target genes.
