# Supplementary material for: Design and function of targeted endocannabinoid nanoparticles
Source: Sci Rep. 2022 Oct 14;12:17260. doi: 10.1038/s41598-022-21715-1 (PMC9568518; doi:10.1038/s41598-022-21715-1)
Supplement: Supplementary file 3 — Supplementary Information 3. [file 41598_2022_21715_MOESM3_ESM.docx]

**SUPPLEMENTARY TABLES**

**TABLE 1.** Raw read and quality metrics of RNA-seq data that were aligned to the GRCh37/hg19 reference genome using STAR

|  | Replicate | Number of reads | Total mapped reads (%) | Uniquely mapped reads (%) | Multi- mapped reads (%) | Per base sequence quality with  score > 30 |
| --- | --- | --- | --- | --- | --- | --- |
| RA-UT | 1 | 10888740 | 98.55% | 84.00% | 14.55% | 50 |
|  | 2 | 11576222 | 98.70% | 84.98% | 13.72% | 50 |
|  | 3 | 11190454 | 98.63% | 84.34% | 14.29% | 50 |
| RA-TNF | 1 | 11449253 | 98.29% | 86.13% | 12.16% | 50 |
|  | 2 | 10455644 | 98.75% | 86.65% | 12.10% | 50 |
|  | 3 | 11921803 | 98.85% | 86.58% | 12.27% | 50 |
| RA-NP | 1 | 11064780 | 98.80% | 84.67% | 14.13% | 50 |
|  | 2 | 11199571 | 98.95% | 84.72% | 14.23% | 50 |
|  | 3 | 11110842 | 98.88% | 84.80% | 14.08% | 50 |
| RA-TNF/NP | 1 | 11602864 | 98.68% | 83.98% | 14.70% | 50 |
|  | 2 | 11649019 | 99.04% | 84.41% | 14.63% | 50 |
|  | 3 | 11903358 | 99.05% | 84.15% | 14.90% | 50 |

**TABLE 2.** Top 10 DE genes based on comparison of RNA-seq data between TNF-α treated (RA-TNF) and non-treated (RA-UT) RA-FLS cells.

| **Gene** | **Description** | **Log2 FC** | **FDR** | **Type(s)** |
| --- | --- | --- | --- | --- |
| **Top 10 DE genes** | | | | |
| IBSP | integrin binding sialoprotein | 13.357 | 1.88 x10^- 28^ | other |
| CXCR4 | C-X-C motif chemokine receptor 4 | 12.839 | 1.2 x10^- 25^ | cytokine |
| CXCL5 | C-X-C motif chemokine ligand 5 | 11.767 | 5.19 x10^- 170^ | cytokine |
| CCL20 | C-C motif chemokine ligand 20 | 11.706 | 3.92 x10^- 57^ | cytokine |
| IL1RN | interleukin 1 receptor antagonist | 11.65 | 7.9 x10- ^22^ | cytokine |
| CXCL8 | C-X-C motif chemokine ligand 8 | 11.523 | 0 | cytokine |
| MMP3 | matrix metallopeptidase 3 | 11.189 | 1.81 x10^- 39^ | peptidase |
| IL23A | interleukin 23 subunit alpha | 10.157 | 1.12 x10^- 16^ | cytokine |
| CXCL10 | C-X-C motif chemokine ligand 10 | 10.155 | 1.25 x10^- 16^ | cytokine |
| C15orf48 | chromosome 15 open  reading frame 48 | 9.887 | 5.55 x10- ^21^ | other |

**TABLE 3.** Top upstream regulators in **(A)** RA-TNF vs RA-UT **(B)** RA-TNF/NP vs RA-TNF.

**A**

| **Gene** | **p-value** | **Status** |
| --- | --- | --- |
| TNF | 4.10 x10^48^ | Activated |
| IFN-γ | 5.97 x10^36^ | Activated |
| RELA | 1.16 x10^28^ | Activated |
| IL-1A | 5.73 x10^25^ | Activated |
| NF-κB | 3.59 x10^23^ | Activated |

**B**

| **Gene** | **p-value** | **Status** |
| --- | --- | --- |
| TNF | 1.70 x10^54^ | Inhibitory |
| IFN-γ | 2.64 x10^48^ | Inhibitory |
| IL1B | 3.29 x10^35^ | Inhibitory |
| lipopolysaccharide | 56.52 x10^34^ | Inhibitory |
| interferon-α | 2.87 x10^31^ | Inhibitory |

**TABLE 4.** Top Canonical Pathways based on comparison of RNA-seq data between **(A)** RA-TNF and RA-UT and **(B)** RA-TNF/NP and RA-TNF

**A**

| **Top canonical pathway** | **p-value** | **overlap** |
| --- | --- | --- |
| Hepatic Fibrosis / Hepatic Stellate Cell Activation | 6.85 x10^11^ | 21.9% (40/183) |
| Granulocyte Adhesion and Diapedesis | 7.65 x10^-10^ | 21.7% (36/166) |
| Dendritic Cell Maturation | 1.34 x10^-09^ | 20.5% (38/185) |
| Agranulocyte Adhesion and Diapedesis | 4.08 x10^-09^ | 20.5% (36/176) |
| Neuroinflammation Signalling pathway | 2.25 x10^-08^ | 16.3% (49/301) |

**B**

| **Top canonical pathway** | **p-value** | **overlap** |
| --- | --- | --- |
| Granulocyte Adhesion and Diapedesis | 3.32 x10^-11^ | 21.1% (35/166) |
| Hepatic Fibrosis / Hepatic Stellate Cell Activation | 3.36 x10-^11^ | 20.2% (37/183) |
| Agranulocyte Adhesion and Diapedesis | 2.93 x10^-09^ | 18.8% (27/121) |
| Role of Macrophages, Fibroblasts and Endothelial Cells in RA | 8.23 x10^-08^ | 14.2% (33/176) |
| LXR/RXR Activation | 1.56 x10^-09^ | 22.3% (43/303) |
